# IRS2 as a driver of brain metastasis in colorectal cancer: A potential target for novel therapeutic strategies

**DOI:** 10.1093/neuonc/noaf028

**Published:** 2025-01-31

**Authors:** Inbal Greenberg, Fayhaa Khair-Dabour, Keren Merenbakh-Lamin, Ethan S Sokol, Anat Klein Goldberg, Dor Simkin, Avishay Spitzer, Moshe Benhamou, Shai Bar-Shira, Michal Raz, Rachel Grossman, Eilam Yeini, Paula Ofek, Tomer Meirson, Ronit Satchi-Fainaro, Hadas Reuveni, Ido Wolf, Tami Rubinek

**Affiliations:** Faculty of Medicine, Tel Aviv University, Tel Aviv, Israel; Department of Oncology, Tel Aviv Sourasky Medical Center, Tel Aviv, Israel; Faculty of Medicine, Tel Aviv University, Tel Aviv, Israel; Department of Oncology, Tel Aviv Sourasky Medical Center, Tel Aviv, Israel; Department of Oncology, Tel Aviv Sourasky Medical Center, Tel Aviv, Israel; Foundation Medicine, Cambridge, MA, USA; Department of Oncology, Tel Aviv Sourasky Medical Center, Tel Aviv, Israel; Department of Oncology, Tel Aviv Sourasky Medical Center, Tel Aviv, Israel; Department of Oncology, Tel Aviv Sourasky Medical Center, Tel Aviv, Israel; Department of Oncology, Tel Aviv Sourasky Medical Center, Tel Aviv, Israel; Faculty of Medicine, Tel Aviv University, Tel Aviv, Israel; Department of Oncology, Tel Aviv Sourasky Medical Center, Tel Aviv, Israel; Institute of Pathology, Tel Aviv Sourasky Medical Center, Tel Aviv, Israel; Brain Tumor Center, Department of Neurosurgery, Rambam Health Care Campus, Rappaport Faculty of Medicine, Technion - Israel Institute of Technology, Haifa, Israel; Faculty of Medicine, Tel Aviv University, Tel Aviv, Israel; Department of Physiology and Pharmacology, Faculty of Medicine, Tel Aviv University, Tel-Aviv, Israel; Department of Physiology and Pharmacology, Faculty of Medicine, Tel Aviv University, Tel-Aviv, Israel; Davidoff Cancer Center, Rabin Medical Center, Petah Tikva, Israel; Sagol School of Neuroscience, Tel Aviv University, Tel Aviv, Israel; Department of Physiology and Pharmacology, Faculty of Medicine, Tel Aviv University, Tel-Aviv, Israel; TyrNovo Ltd. Science Park, Rehovot, Israel; Purple Biotech Ltd. Science Park, Rehovot, Israel; Faculty of Medicine, Tel Aviv University, Tel Aviv, Israel; Department of Oncology, Tel Aviv Sourasky Medical Center, Tel Aviv, Israel; Faculty of Medicine, Tel Aviv University, Tel Aviv, Israel; Department of Oncology, Tel Aviv Sourasky Medical Center, Tel Aviv, Israel

**Keywords:** β-catenin, brain metastasis, colorectal cancer, IRS2, oxidative phosphorylation

## Abstract

**Background:**

Colorectal cancer (CRC) ranks as the fourth most common cause of brain metastasis (BM), with its incidence on the rise. However, the molecular mechanisms driving the formation of these lesions from CRC remain unclear.

**Methods:**

We analyzed the Foundation Medicine genomic database, which includes over 35,000 CRC samples from both local and metastatic sites. The role of insulin receptor substrate 2 (IRS2) in CRC brain tropism was investigated using various in vitro (co-culture systems and 3D sphere formation assays), in vivo (intracranial and subcutaneous mouse models), and ex vivo (CRC Patient-Derived Explants) models. The molecular and metabolic effects of IRS2 were examined through RNA sequencing and Seahorse analysis. The therapeutic potential of a combined treatment with NT219, an IRS2 inhibitor, and 5-fluorouracil (5-FU) was assessed using our CRC BM mouse model.

**Results:**

Our research reveals a distinctive genomic profile of CRC BM and highlights the role of IRS2 in promoting CRC BM. IRS2 mediates its effect by modulating the β-catenin and oxidative phosphorylation (OXPHOS) pathways. We developed a mouse model of BM from CRC and demonstrated that treatment with the IRS2 inhibitor NT219, in combination with 5-FU, significantly suppresses BM development and prolongs survival.

**Conclusions:**

Our work underscores the unique role of IRS2 in facilitating CRC brain adaptation and suggests a novel therapeutic strategy for CRC patients with BM.

## Graphical Abstract

**Graphical Abstract:**
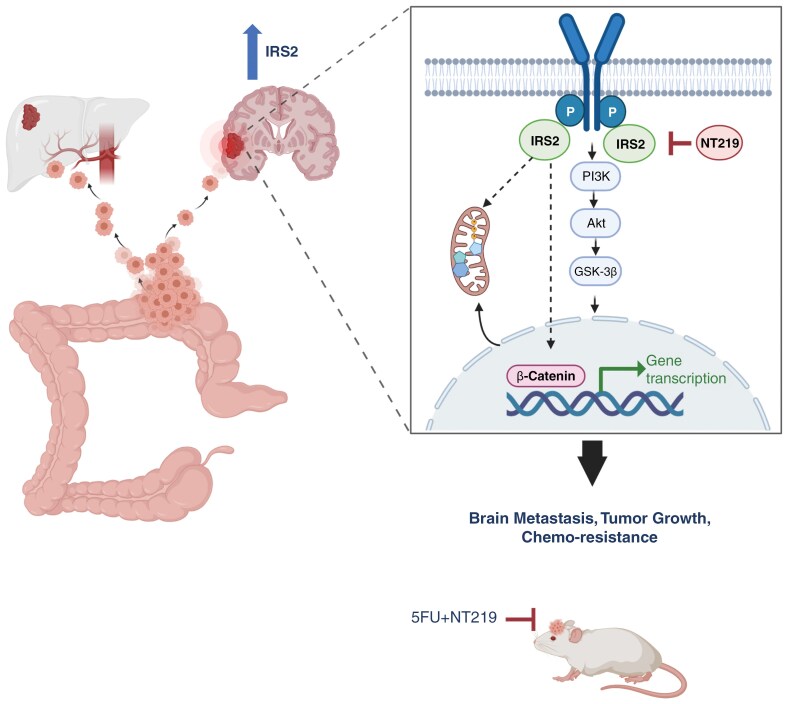

**Key Points**

- IRS2 amplification and expression are increased in colorectal cancer (CRC) brain metastasis.
- IRS2 enhances CRC cells survival in the brain by altering β-catenin and mitochondrial activity.
- Combination therapy of NT219 and 5-fluorouracil inhibits CRC brain metastasis and extends survival.

**Importance of the Study**

Colorectal cancer (CRC) has become the fourth leading cause of brain metastases (BM), yet the mechanisms driving CRC BM formation remain largely elusive. This study presents the most extensive genomic analysis of over 35,000 CRC samples, shedding light on the biology of CRC BM. The research identifies a distinct genomic profile associated with CRC BM and highlights the role of insulin receptor substrate 2 (IRS2) in promoting BM formation. We show that IRS2 facilitates CRC BM through modulation of the β-catenin pathway and oxidative phosphorylation. Importantly, the study demonstrates that combining 5-fluorouracil with NT219, an IRS2 inhibitor currently in early-phase clinical trials, significantly impedes BM development and extends survival rates. These findings advocate for the utilization of IRS2 inhibitors as a promising therapeutic strategy against CRC BM, offering potential avenues for improved treatment strategies.

CRC is the third most diagnosed cancer and the second-leading cause of cancer-related deaths worldwide.^1^ Around 20% of CRC patients have distant metastases at diagnosis, and another 50% develop metastases later, with the liver being the most common site.^2^ However, as treatments improve and patients live longer, the incidence of CRC BM is rising, now the fourth leading cause of BM.^3^ CRC BM has a poor prognosis, with a median survival of just 5.3 months, highlighting the urgent need for effective therapies.

Metastasis to the brain requires cancer cells to adapt to a unique environment, including penetrating the blood-brain barrier (BBB) and surviving low oxygen and nutrient levels. Understanding these adaptations can reveal targets for new treatments.^4^

While the genomic alterations (GA) in primary CRC and liver or lung metastases are well understood, those driving CRC BM are not.^5^ Other cancers that metastasize to the brain, like lung, melanoma, and breast cancers, commonly show alterations in the PI3K/AKT/mTOR pathway.^6^ Therefore, we hypothesized that specific GA promote CRC BM.

IRS2 is a cytoplasmic adaptor protein that mediates the activity of insulin and IGF-1 receptors, activating the downstream PI3K/AKT/mTOR and MAPK pathways.^7^ IRS2 is expressed in various malignancies and contributes to unique tumor cell metabolism, motility, and invasion.^8^ In CRC, IRS2 mRNA and protein levels increase with progression from normal tissue to adenoma and carcinoma and dysregulated IRS2 expression activates the PI3K/AKT/mTOR pathway and enhances cell adhesion.^9^

Our analysis of a genomic database of over 35,000 CRC biopsies obtained from both local and metastatic sites, shows higher IRS2 gene amplification in CRC BM compared to other sites. IRS2 supports CRC cell survival in the brain environment through transcriptional and metabolic changes, as evidenced by in vitro and in vivo studies. Furthermore, combining the IRS2 inhibitor NT219 with the chemotherapy 5-FU significantly reduces CRC BM development. Collectively, our study identifies IRS2 as a key factor in promoting CRC brain adaptation and suggests a new therapeutic strategy for CRC BM patients.

## Methods

### Ethical Statement

Approval for the Foundation Medicine dataset portion of the study was obtained from the Western Institutional Review Board (Protocol No. 20152817).

Patient-derived CRC BM, LM and primary tissues were collected after written informed consent was obtained from the research subjects by the Tel Aviv Sourasky Medical Center, under an approved institutional review board (IRB) (0172-17-TLV).

Mice were maintained at the SPF facilities of Tel Aviv University. All experiments involving animals were approved by the TAU Institutional Animal Care and Use Committee (approval 01-19-007).

### Comprehensive Genomic Profiling

Comprehensive genomic profiling was performed in a CLIA-certified, CAP-accredited laboratory (Foundation Medicine Inc., MA, USA) on CRC all-comers during routine clinical care. Hybrid capture was carried out on exons from up to 395 cancer-related genes (FoundationOne v3: 323; FoundationOne v5: 395) and select introns from up to 31 genes frequently rearranged in cancer (FoundationOne v3: 24; FoundationOne v5: 31) (Supplementary Table S1 and Supplementary Table S2). See Supplementary Methods for details.

### CRC Patient-Derived Explant (PDE)

PDE involve the culture of resected tumor fragments that retain the TME native architecture, tumor heterogeneity, immune profile, and proliferative capacity.^10^ See Supplementary Methods for details.

### Cells and Transfections

The human primary CRC cell lines HCT116, HT29, SW480, SW403, and LS513, and the human embryonic kidney cell line HEK293T were obtained from the ATCC and authenticated with ATCC DNA markers. Human astrocytes (ScienCell Research Laboratories), human microglia (Celprogen), human lung-derived small airway epithelial cells and human hepatocytes (Lonza) were grown and maintained according to provider instructions. See Supplementary Methods for details.

### Chemicals

See Supplementary Methods for details.

### Conditioned Media

Human astrocytes, microglia, lung-derived small airway epithelial cells, and hepatocytes were cultured to confluency in T75 flasks. Cells were washed three times with PBS to remove residual growth medium and incubated in fresh serum-free medium (SFM) for 24 hours. Conditioned media (CM) was collected, filtered using 0.2 µm filters (Millipore), and used fresh for each experiment. SFM served as the control.

### Generation of Stable Cells

#### IRS2 overexpression.

pReceiver-Lv247-IRS2 (GeneCopoeia; GC-EX-Y3534-Lv247) or pReceiver-Lv247-Empty control (GeneCopoeia; GC-EX-NEG-Lv247) vectors were transfected along with pCMV-VSV-G (Addgene; 8454) and psPax2 (Addgene; 12260) (2 µg: 2 µg: 2 µg) into HEK-293T cells using PEI reagent. CM was collected 48 and 72 hours post-transfection, filtered, and supplemented with polybrene (8 µg/ml) to infect HCT116, HT29, and SW480 cells for 8 hours. Stably infected cells were selected with puromycin (0.75 µg/ml), and single colonies were isolated and assessed for IRS2 expression. Two clones with the highest IRS2 expression were cultured routinely separately and mixed before experiments.

#### IRS2 silencing.

HEK-293T cells were transfected with short hairpin RNA (shRNA) targeting IRS2 (_sh-IRS2_, Sigma; TRCN0000061257), IRS2-2 (_sh-IRS2_, Sigma; TRCN0000312551) or nonspecific control (^sh-NS^, Sigma; TRCN0000364328) vectors along with pCMV-VSV-G and psPax2, as described above. SW403 and LS513 cells were infected and clones with the lowest IRS2 expression were isolated as described above.

### Intracranial Mouse Model

Six-week-old male athymic nude mice (Envigo CRS, Israel) were anesthetized with ketamine (150 mg/kg) and xylazine (12 mg/kg) intraperitoneally. A 1-cm incision was made on the skull midline between the ears, and a small hole was drilled 2 mm left, 0.5 mm anterior and 6 mm ventral to the bregma. HCT116^CON/^^IRS2^ or LS513^sh-NS/^^sh-IRS2^ cells (2 × 10^5^ cells) were inoculated, and the incision was closed by wound clips.

For the NT219 and 5-FU experiment, LS513^sh-NS^ cells (2 × 10^5) were implanted, and mice were treated with 5-FU (30 mg/kg, *n* = 9), NT219 (70 mg/kg, *n* = 9), combined treatment (*n* = 8), or control vehicle (*n* = 9), intraperitoneally twice a week.

Animals were monitored twice a week for general health and body weight. Tumor development was followed by MRI (T1 weighted with contrast agent; Bruker, Germany). Mice were euthanized when they lost 10% of body weight in a week, 20% of their initial weight, or when neurological symptoms appeared.

### Subcutaneous Mouse Model, MRI and Immunofluorescence

See Supplementary Methods for details.

### Immunohistochemistry (IHC)

Immunohistochemistry staining was performed on FFPE CRC BM or LM tissue using a monoclonal anti-IRS2 antibody (ab134101; Abcam). See Supplementary Methods for details.

### RNA Sequencing (RNA-seq) and Analysis

#### RNA-seq.

SW403^sh-NS/sh-IRS2^ and HCT^CON/IRS2^ cells were seeded in triplicate for 24 h. Total RNA was extracted using the High Pure RNA Isolation Kit (Roche). The libraries were prepared using NEBNext® Ultra™ II RNA Library Prep Kit for Illumina® (New England BioLabs® Inc.). Libraries were evaluated by Qubit and TapeStation. Sequencing was conducted with NextSeq 500/550 v2.5 (Illumina) at 75-cycles, Single Read kit. The output was ~21 million reads per sample. See Supplementary Methods for details.

#### Analysis.

Genes that were differentially expressed between the comparison and control groups were characterized using R package DESeq2^11^ by identifying genes with |log2FC|>1 and a *P*-adjusted value < 0.05 (Supplementary Table S3). Pathway analysis of the differentially expressed genes (DEGs) was performed using the R package clusterProfiler.^12^ To gain an initial functional understanding of the data, we conducted an overrepresentation analysis (ORA) using the hypergeometric statistical test to assess significance (FDR; Benjamini–Hochberg procedure < 0.05) (Supplementary Table S4). Genes determined to be DEGs were compared with gene sets collected from the Human Molecular Signatures Database (MSigDB) “Hallmark” and “Curated Canonical KEGG” on the background of universe genes consisting of all genes expressed in the samples.

STRING database analysis^13^ was done to examine the role of DEGs in known and predicted protein–protein interactions.

To complement these analyses and reveal a more subtle yet coordinated change in the expression of genes that share the same functional pathway, we employed gene-set enrichment analysis (GSEA)^14^ (Supplementary Table S5). For the GSEA analysis, the same gene sets were used as for the ORA. To conduct a targeted examination of metabolic alterations, we manually curated a collection of 70 metabolic pathways from the KEGG database and analyzed these gene sets for enrichment^15^ (Supplementary Table S6).

### Seahorse Analysis

Oxygen consumption rate (OCR) and extracellular acidification rate (ECAR) were measured with a Seahorse XF96 Analyzer (Agilent) following the manufacturer’s instructions. Mitochondrial respiration was assessed using the Seahorse Cell Mito Stress Test kit, and glycolytic activity was evaluated using the Seahorse Glycolysis Stress Test kit. See Supplementary Methods for details.

### Quantitative RT-PCR (qRT-PCR)

Total RNA extraction, cDNA synthesis, and (qRT-PCR) were conducted as previously described.^16^ Primers (IDT) are listed in Supplementary Table S7.

### Western blot, Colony Assay, Methylene Blue Assay, Invasion Assay, Migration (Transwell) Assay, 3D Sphere Formation Assays, Luciferase Assay and Growth Factors Analysis

See Supplementary Methods for details.

### Drug Interaction Analysis

*The Bliss Independence method* was employed to determine drug interaction.^17^ See Supplementary Methods for details.

### Graphical Illustrations

Graphical elements for the experimental design schemes were created using Biorender, licensed by the Tel Aviv University Medical Faculty.

### Statistical Analysis

Statistical analyses were performed using GraphPad Prism 10.2. See Supplementary Methods for details.

## Results

### IRS2 Amplification is Increased in CRC BM

**Figure 1.**
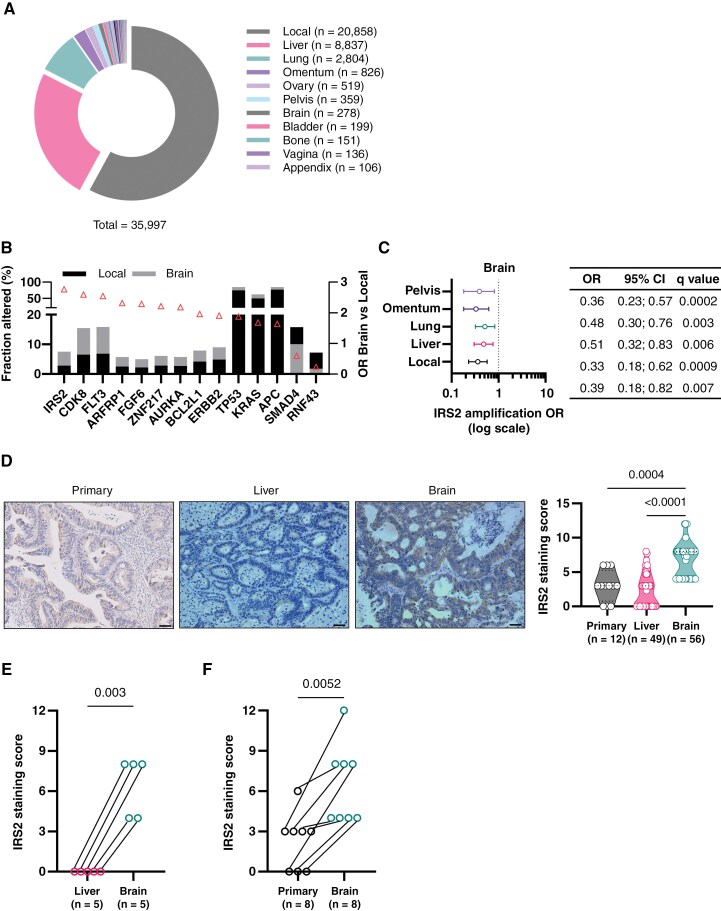
IRS2 amplification is increased in CRC BM. (A) Distribution of 35,997 CRC clinical samples, analyzed for GA, according to the biopsy site. (B) The graph depicts the most significantly altered genes in CRC BM compared to the local. Triangle represents the odds ratio (OR). (Fisher’s exact test with FDR-corrected *P*-value). (C) IRS2 amplification in CRC BM compared to local and different metastatic sites (Fisher’s exact test with FDR-corrected *P*-value). (D) Representative IRS2 IHC staining of CRC primary (n = 12), unmatched LM (*n* = 49), and unmatched BM (*n* = 56). Scale bars represent 50 µm. Quantification of IRS2 staining was performed using the immuno-reactive score (IRS) (Kruskal-Wallis one-way ANOVA). (E) IRS2 immunostaining of CRC LM and BM. The institutional cohort contained five matched LM and BM samples. Depicted the IRS2 IHC staining score of CRC BM (*n* = 5) and matched LM (*n* = 5) (paired t test, two-tailed). (F) IRS2 immunostaining of CRC primary and BM. The institutional cohort contained eight matched primary and BM samples. Depicted the IRS2 IHC staining score of CRC BM (*n* = 8) and matched primary (*n* = 8) (paired t test, two-tailed).

In order to decipher the molecular landscape of CRC BM, we analyzed the GA prevalence in CRC clinical samples from local (*n* = 20,858) and metastatic (*n* = 15,139) sites using a large real-world database (Foundation Medicine Inc.). The majority of metastatic samples originated from the liver (*n* = 8,837; 58%), followed by lung (*n* = 2,804; 19%), omentum (*n* = 826; 5%), and brain (*n* = 278; 2%) (Figure 1A). Patients with BM biopsies were similar in age and sex to patients with biopsies from other sites (median 60 vs 60 respectively, *P* = 0.16; male 57.97% vs 53.43% respectively, *P* = 0.1). Median tumor mutational burden was modestly higher in BM samples compared to other metastatic sites (5.0 vs 3.8, respectively, *P* < 3E−11) (Supplementary Table S8). Compared to local tumors, BM exhibited a higher incidence of GA in several genes, including IRS2, CDK8, FLT3, and KRAS (Figure 1B and Supplementary Figure S1A–D). Among these, IRS2 gene showed the highest odds ratio (OR) (2.8 OR; *P* = 0.001), occurring in 7.6% (21/278) of BM compared to 2.9% (598/20,858) of local tumors (Figure 1B). The majority of GA in the IRS2 gene were amplification, observed across all sample types (Supplementary Figure S1E). This increase in IRS2 amplification was particularly specific to BM, as other metastatic sites demonstrated a lower OR compared to BM (Figure 1C). Notably, significant enrichment of IRS2 was exclusive to BM from CRC and was not observed in BM from other primary tumors when compared to their local tumors, including lung cancer (0.8% vs 0.6%, *P* = 0.4), breast cancer (2.6% vs 1.5%, *P* = 0.1), and melanoma (0.2% vs 0.3%, *P* = 1) (Supplementary Table S9). Moreover, we conducted a correlation analysis between IRS2 amplification and routine biomarkers (KRAS, NRAS, BRAF, ERBB2, APC, and TP53) in CRC patients (Supplementary Table S10). In BM, IRS2 amplification significantly co-occurred with NRAS alterations (OR 5.04, *P* = 0.02) (Supplementary Table S10).

To further validate the hypothesis connecting IRS2 amplification with an increased risk of BM in CRC, we analyzed the MSK-MET tropism cohort,^18^ comprising 3,548 CRC samples from primary tumors (*n* = 2,401) and metastases (*n* = 1,147), including 22 BM samples (Supplementary Figure S1F). IRS2 amplification was significantly more common in BM, observed in 18% (4/22) of cases, compared to 1.5% (37/2,401) in primary tumors (*P* = 0.0004) and 2% (23/1,125) in non-BM (*P* = 0.001) (Supplementary Figure S1G and S1H). These findings support the association between IRS2 amplification and CRC BM.

We were intrigued by IRS2, as its downstream signaling pathway has previously been associated with BM development via PTEN loss.^19^ Furthermore, the three key altered genes - IRS2, CDK8, and FLT3 - are all located on chromosome 13q, a region commonly gained in CRC with a prevalence of up to 85%.^20^ A recent study analyzing 124 primary CRCs refined the minimal copy region on 13q and identified IRS2 as the only whole gene within it, supporting its role as a potential driver oncogene.^9^

In order to validate these data and corroborate the association between the genomic data and protein expression, we examined the IRS2 expression using IHC staining of human CRC samples from BM (*n* = 56), liver metastasis (LM) (*n* = 49), and primary tumor (*n* = 12). Increased IRS2 expression was noted in BM compared to LM (*P* < 0.0001) and primary tumor samples (*P* = 0.0004) (Figure 1D and Supplementary Figure S2A and B). No significant differences were noted between BM and LM groups for age, sex, or primary tumor location (Supplementary Table S11). Notably, IRS2 expression was consistently higher in BM compared to matched samples of primary tumors and LM (Figure 1E and F).

Finally, we analyzed mortality data for patients with BM (*n* = 56), comparing those with low IRS2 expression (IRS score ≤ 6, *n* = 24) to those with high IRS2 expression (IRS score > 6, *n* = 32). During a median follow-up of 1.1 years, all-cause mortality occurred in all 56 (100%) patients with BM. Kaplan-Meier curves demonstrated significantly higher mortality in patients with high expression (log-rank, hazard ratio (HR) 1.75, 95% confidence interval (CI) 1.03–2.96, *P* = 0.028) (Supplementary Figure S2C). In multivariate Cox regression analysis, after adjusting for age at diagnosis, sex, and primary CRC location, IRS score remained significantly associated with increased mortality (HR 1.3, 95% CI 1.1–1.6, *P* = 0.005) (Supplementary Figure S2D).

Therefore, we focused our further investigation on how high IRS2 expression levels promote the development of CRC BM.

### IRS2 Enhances Tumorigenicity of CRC Cells Within the Brain Environment

**Figure 2.**
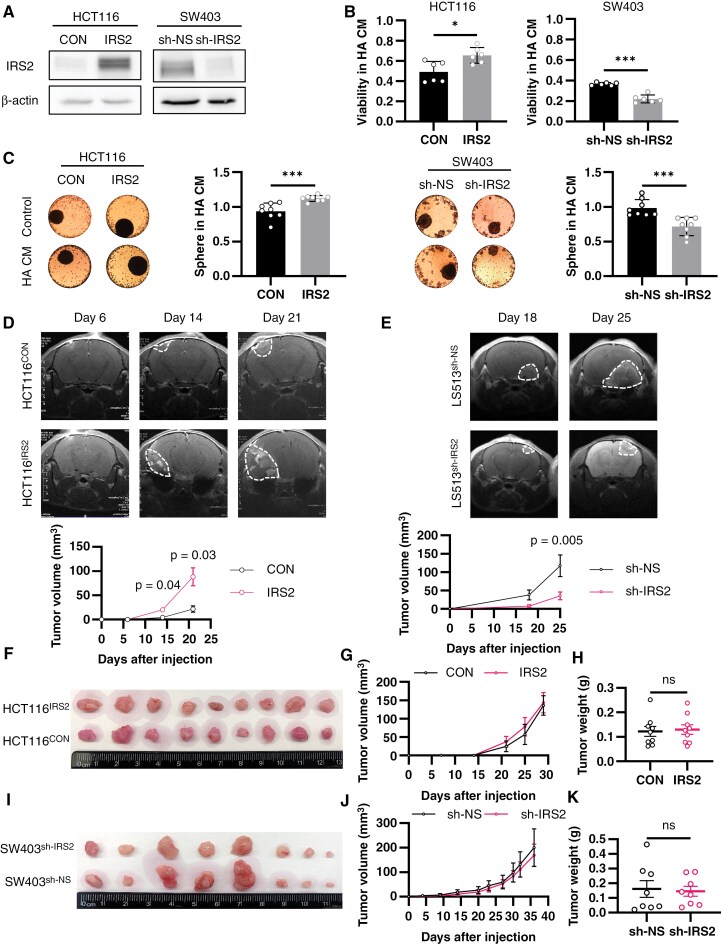
IRS2 enhances tumorigenicity of CRC cells within the brain environment. (A) HCT116 cells were infected with pReceiver-Lv247-IRS2 (IRS2) or pReceiver-Lv247-Empty control (CON), and SW403 cells were infected with shRNA against IRS2 (sh-IRS2) or nonspecific sequence control (sh-NS). IRS2 protein levels were determined by western blot and normalized to β-actin level. (B) Cells were seeded in HA CM or control (HA SFM) using inSphero assay. As those conditions did not support 3D sphere generation, we utilized the assay for 2D culture viability. Viability was evaluated by Realtime-Glow MT and normalized to the control (HA SFM) (two-way ANOVA). (C) Spheres were generated, and medium was changed to HA CM or control (HA SFM). Viability was evaluated by Realtime-Glow MT, normalized to the control (HA SFM), and spheres were photographed after 72 h (two-way ANOVA). (D) Intracranial CRC BM mouse model was employed using HCT116^CON^ (*n* = 10) or HCT116^IRS2^ cells (*n* = 7). MRI T1-weighted gadolinium contrast was conducted on d 6, 14, and 21 post cells implantation. Representative MRI images of one mouse brain from each group are shown. Tumor volume was quantified using MRIcro software, error bars represent s.e.m. (repeated measures ANOVA). (E) The same BM model as in (D) was employed using LS513^sh-NS^ or LS513^sh-IRS2^ cells (*n* = 7, per group). Tumors were imaged and quantified similarly on d 18 and 25 post cells implantation, error bars represent s.e.m. (repeated measures ANOVA). (F) Gross images of xenograft tumors derived from subcutaneous implantation of HCT116^CON^ or HCT116^IRS2^ cells (n=9, per group). (G) Tumor growth curve measured manually by caliper, error bars represent s.e.m. (repeated measures ANOVA). (H) Tumor weight at time of resection, dots represent individual mice, error bars represent s.e.m. (unpaired t test, two-tailed). (I) Gross images of xenograft tumors derived from subcutaneous implantation of SW403^sh-NS^ or SW403^sh-IRS2^ cells (n=8, per group). (J) Tumor growth curve measured manually by caliper, error bars represent s.e.m. (repeated measures ANOVA). (K) Tumor weight at time of resection, dots represent individual mice, error bars represent s.e.m. (unpaired t test, two-tailed).

IRS2 protein serves as an adaptor in the insulin and IGF signaling cascades, which are crucial for regulating tumor progression and metabolism.^8^ We first characterized IRS2 mRNA and protein levels in three IRS2 non-amplified (HCT116, HT29, and SW480) and two IRS2-amplified (SW403 and LS513) CRC cell lines.^21^ As expected, a correlation between IRS2 mRNA (Supplementary Figure S2E) and protein (Supplementary Figure S2F) expression and IRS2 copy number was observed. In order to test the impact of IRS2 on CRC cells, we either overexpressed IRS2 in the IRS2 non-amplified cell lines (denoted HCT116_IRS2_ HT29_IRS2_ and SW480_IRS2_), or silenced it in the IRS2-amplified cell lines (denoted SW403_sh-IRS2_ SW403_sh-IRS2_ and LS513_sh-IRS2_) (Figure 2A and Supplementary Figure S2G) and assessed their oncogenic characteristics. Cells overexpressing IRS2 exhibited increased proliferation, colony formation, migration, invasion, and 3D sphere formation, while IRS2-silenced cells showed the opposite trend (Supplementary Figure S3A–E).

Astrocytes, the most abundant glial cells unique to the brain microenvironment, have been shown to support tumor cell proliferation, invasion, and metastasis, while also protecting them from the cytotoxic effects of chemotherapy.^22^ Hence, we aimed to explore the effect of IRS2 on CRC cells in their adaptation and interaction with astrocytes. Cells overexpressing IRS2 demonstrated enhanced viability and sphere formation in human astrocyte-conditioned media (HA CM) or co-culture with HA while IRS2-silenced cells displayed reduced viability and sphere formation (Figure 2B and C and Supplementary Figure 4A–C). To directly address the effects of IRS2 on CRC brain adaptation in vivo, IRS2-manipulated CRC cells were injected into the brains of athymic nude mice, and tumor growth was measured by MRI. Consistent with the in vitro experiments, HCT116^IRS2^ cells formed larger tumors and exhibited accelerated growth (by 304% 21 days after injection, *P* = 0.03) (Figure 2D), whereas, LS513^sh-IRS2^ cells showed decreased tumor outgrowth (by 247% 25 days after injection, *P* = 0.005) (Figure 2E).

Subsequently, we sought to determine whether the growth advantage conferred by IRS2 in the presence of HA extends to other environments. To this end, we used CM from microglia cells, representing another brain environment component, and from lung-derived small airway epithelial (lung) cells, a common CRC metastatic site. Additionally, we evaluated if this advantage persists in vivo using a subcutaneous model. IRS2-expressing cells retained their growth advantage when exposed to microglia CM (Supplementary Figure S4D and E) but not to lung CM (Supplementary Figure S5A and B) or in the in vivo subcutaneous tumor model (Figure 2F–K).

Finally, to obtain a more comprehensive understanding of the unique factors secreted by HA, we conducted a growth factors analysis. Out of the 40 growth factors examined, we observed upregulation of insulin, VEGF, HGF, and IGFBP-2 (Supplementary Figure S5C). These factors have been previously associated with the brain environment.^23^ Notably, insulin and IGFBP-2 are critical regulators of IRS2.^24^ Among the four factors significantly increased in HA CM compared to human hepatocytes (liver) or lung CM, insulin uniquely showed an exclusive rise in HA CM (Supplementary Figure S5D). Collectively, these data indicate growth advantage for IRS2-expressing CRC cells specifically in the brain microenvironment.

### IRS2 Enhances Metastasis-Associated Genes Expression

**Figure 3.**
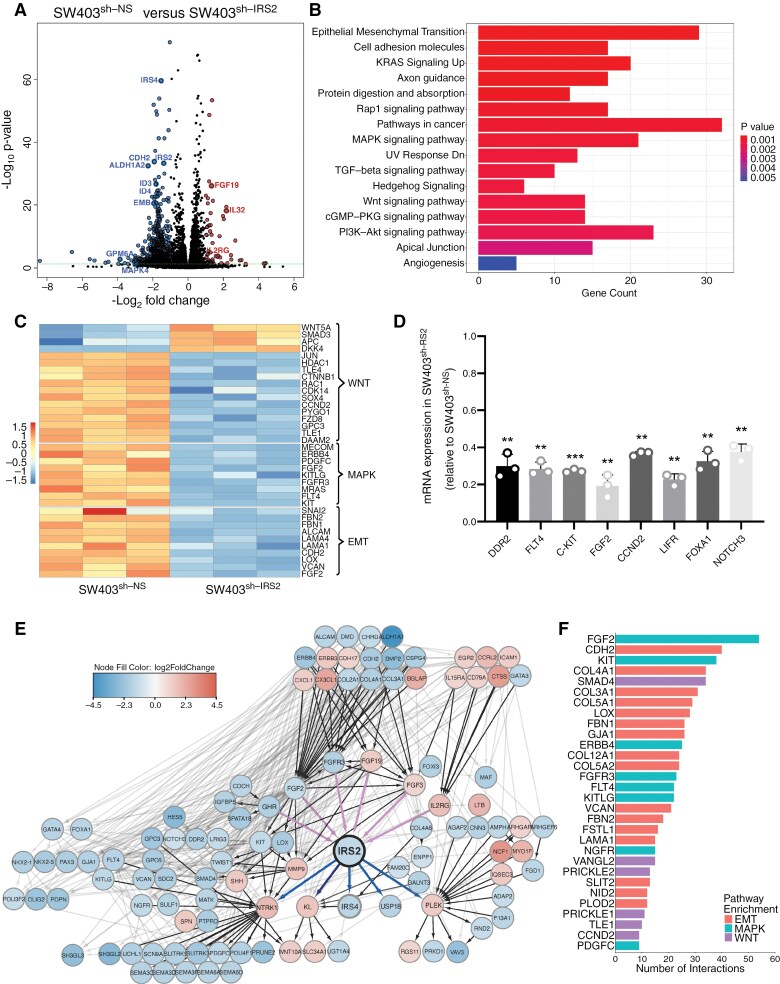
IRS2 enhances aggressiveness-associated gene signatures in CRC Cells. Transcriptomic analysis of SW403^sh-NS^ or SW403^sh-IRS2^ cells grown under standard conditions was performed using RNAseq. (A) Volcano plot depicting differentially expressed genes (DEGs) between SW403^sh-NS^ and SW403^sh-IRS2^ cells. Each circle represents a gene. Significant DEGs are defined by a fold change of ≥2 (Log2FC ≥1) and an adjusted P-value <0.05. Dots on the left indicate downregulated genes, while dots on the right indicate upregulated genes. (B) Barplot visualization of all significantly over-represented MSigDB curated “Hallmark” and “Canonical KEGG” gene sets in SW403^sh-NS^ versus SW403^sh-IRS2^. The color of the bars represents the *P* value for each enriched gene set identified by Fisher’s exact test. The bar length represents the number of differentially expressed genes enriched in each gene set. (C) Heatmap of significantly differentially expressed genes (*P*-value < 0.05) grouped by their respective function in SW403^sh-IRS2^ compared with SW403^sh-NS^ cells. (D) Validation of chosen downregulated genes related to metastasis formation in SW403^sh-IRS2^ compared to SW403^sh-NS^ cells by qRT-PCR (two-way ANOVA). (E) Protein–protein interactions among products of 652 DEGs (|log_2_FC|>1, Benjamini–Hochberg-adjusted *P* value < 0.05), analyzed using the search tool for retrieval of interacting genes or proteins (STRING) database. A graph was constructed using Cytoscape software and filtered to represent only IRS2 interacting cluster. Each node is colored by z-normalized expression value and the edges represent its associated protein interactions. (F) Bar plot showing the number of interactions for the top 30 most interacting proteins within the IRS2-interacting cluster. Bars are categorized based on associated KEGG pathways, including EMT, MAPK, and WNT signaling.

In order to gain insight into the mechanism underlying the aggressive phenotype of IRS2-expressing cells, we performed global transcriptomic analysis using RNA-seq, comparing SW403^sh-IRS2^ cells to SW403^sh-NS^ cells. We noted 652 DEGs (pFDR < 0.05 and |log_2_FC|>1) (Supplementary Table S3), with 90% being downregulated in SW403^sh-IRS2^ cells (Figure 3A). Pathway enrichment analysis uncovered significant alterations in pathways associated with adhesion, angiogenesis, and cancer-related processes, primarily characterized by a decrease in genes linked to aggressiveness in SW403^sh-IRS2^ cells (Figure 3B and Supplementary Table S4). Genes affected by IRS2 silencing encompassed molecules from the WNT/β-catenin signaling pathway (e.g. DKK4 and CCND2), epithelial–mesenchymal transition (EMT) process (e.g. LOX and FGF2), and MAPK signaling pathway (e.g. KIT and ERBB4) (Figure 3C). qRT-PCR analysis confirmed reduced expression of aggressiveness and metastasis-related genes in SW403^sh-IRS2^ cells compared to SW403^sh-NS^ cells (Figure 3D and Supplementary Figure S6A). The opposite trend was observed in HCT116^IRS2^ cells (Supplementary Figure S6B). Similar results were also observed under HA CM conditions (Supplementary Figure S6C–E). Protein–protein interaction analysis of the 652 DEGs with a focus on the IRS2 interacting cluster showed subnetworks of interrelated proteins (Figure 3E), with prominent nodes involving the WNT/β-catenin pathway molecules (e.g. SMAD4 and VANGLT2), EMT process markers (e.g. CDH2 and COL4A1), and MAPK pathway molecules (e.g. FGF2 and KIT) (Figure 3F).

Comparison of HCT116^CON^ and HCT116^IRS2^ cells using identical experimental and analytical methodologies demonstrated significant concordance with the findings from SW403 cells (Supplementary Figure S6F, Supplementary Table S12). Pathway analysis revealed notable alterations linked to IRS2 overexpression in pathways associated with tumor aggressiveness, including extracellular matrix remodeling, cell adhesion, and locomotion (Supplementary Figure S6G, Supplementary Table S13). Furthermore, the similarity between gene sets enriched in IRS2 silencing and overexpression experiments was significantly greater than expected by chance, with an observed Jaccard similarity index of 0.24 compared to 0.12 expected by chance (*P* = 0.002, bootstrapping).

These findings indicate potential mechanisms driving the aggressive phenotype of IRS2 in CRC cells.

### Increased IRS2 Levels Are Associated With Enhanced Mitochondrial Activity of CRC Cells

**Figure 4.**
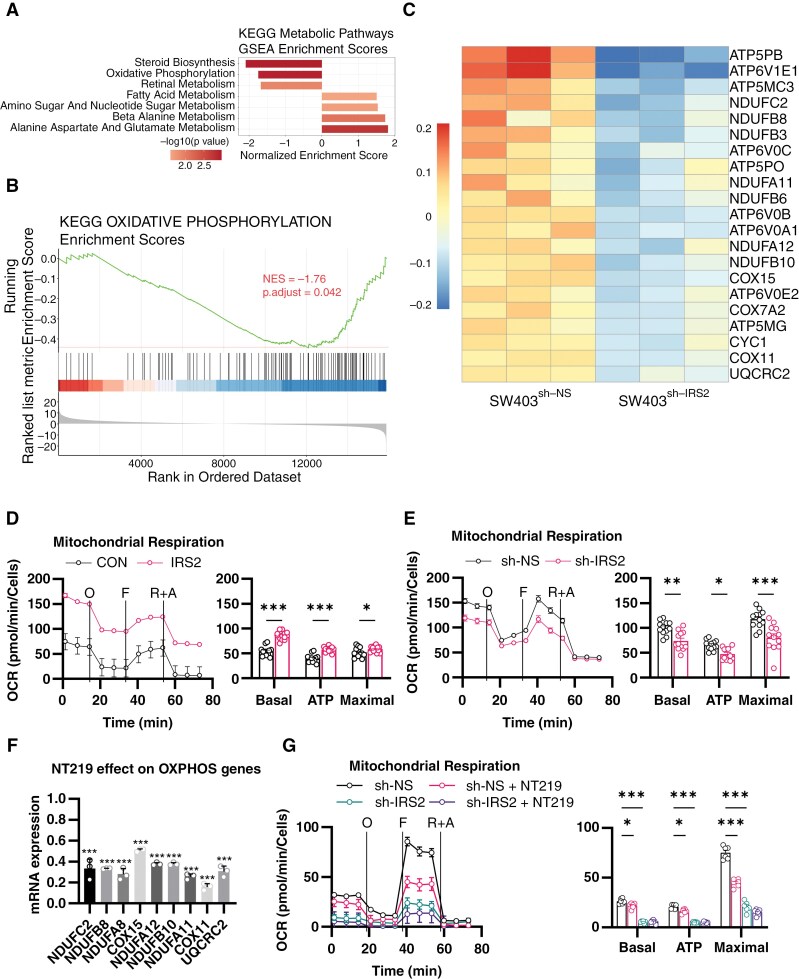
IRS2 increases mitochondrial activity in CRC cells. (A) Pre-ranked GSEA demonstrating significantly altered KEGG metabolism pathways (*P* value < 0.05, FDR & q-values < 0.25) in SW403^sh-IRS2^ compared with SW403^sh-NS^ cells. The normalized enrichment score (NES) forms the x-axis. (B) GSEA enrichment plot showing significant negative NES of the KEGG OXPHOS gene set in SW403^sh-IRS2^ compared with SW403^sh-NS^ cells. NES and FDR (Benjamini–Hochberg method) are listed on the plot. (C) Heatmap of significantly differentially expressed genes (*P*-value < 0.05) related to OXPHOS in SW403^sh-IRS2^ compared with SW403^sh-NS^ cells. (D and E) Mitochondrial respiration was studied by monitoring oxygen consumption rate (OCR) using Seahorse Cell Mito Stress Test kit. (D) HCT116^CON^ or HCT116^IRS2^ or (E) SW403^sh-NS^ or SW403^sh-IRS2^ were seeded and OCR was measured under basal conditions followed by the sequential addition of oligomycin (O), FCCP (F), and rotenone and antimycin A (R + A). Bar chart showing basal respiration, ATP production and maximal respiration; Error bars represent s.e.m. (two-way ANOVA). (F) LS513 cells were treated with NT219 (5 µM) or control vehicle (D5W). Expression of genes related to OXPHOS was determined by qRT-PCR (two-way ANOVA). (G) LS513^sh-NS^ or LS513^sh-IRS2^ were treated with NT219 (5 µM) or control vehicle (D5W) for 4 h and OCR was assessed as described above (D and E); error bars represent s.e.m. (two-way ANOVA).

IRS2 serves as a crucial hub in insulin/IGF-1-signaling, regulating metabolic processes in cancer cells.^8^ Given the distinct conditions in the brain microenvironment compared to the colon and other metastatic sites, which include lower oxygen levels and reduced glucose availability,^25^ we postulated that IRS2 facilitates the metabolic adaptation of CRC cells to the brain. We first selected 70 MSigDB KEGG metabolism-specific gene sets and performed GSEA-*P* on the transcriptomic analysis comparing SW403^sh-IRS2^ cells to SW403^sh-NS^ cells under standard growth conditions. The analysis identified OXPHOS as one of the pathways significantly downregulated upon IRS2 silencing (Figure 4A and B and Supplementary Table S6). Conversely, in a similar transcriptomic analysis of HCT116 cells, OXPHOS levels were upregulated with IRS2 overexpression (Supplementary Table S14).

Using the transcriptomic data, we generated a heat map of the 21 significantly differentially expressed OXPHOS-associated genes (*P*-value < 0.05) and noted down-regulation of all these genes in SW403^sh-IRS2^ cells (Figure 4C). The majority of these genes were related to respiratory complex I—NADH dehydrogenase (Supplementary Figure S7A). qRT-PCR analysis confirmed reduced expression of OXPHOS-associated genes in SW403^sh-IRS2^ cells compared to SW403^sh-NS^ cells (Supplementary Figure S7B and C). The same trend was observed in LS513^sh-IRS2^ cells and the opposite in HCT116^IRS2^ cells (Supplementary Figure S7D and E).

In order to elucidate the association between IRS2 expression and mitochondrial activity, we first measured mitochondrial respiration and glycolysis by Seahorse extracellular flux analysis in HCT116^IRS2^ cells or SW403^sh-IRS2^ cells compared to control cells. HCT116^IRS2^ cells exhibited increased basal mitochondrial respiration, ATP production, and maximal respiration compared to control cells (Figure 4D), while the opposite was observed in SW403^sh-IRS2^ cells (Figure 4E). Glycolysis, glycolytic capacity, and glycolytic reserve were, however, unaffected by IRS2 expression in both cell types (Supplementary Figure S8A and B). Additionally, IRS2-expressing cell viability was less affected by 2-DG, a glucose analog that inhibits glycolysis (Supplementary Figure S8C and D).

To further investigate the role of IRS2 in promoting this metabolic shift, we employed the first-in-class IRS2 inhibitor, NT219, which triggers serine phosphorylation and degradation of IRS2^26^ while also suppressing STAT3 through dephosphorylation.^27^ Treatment with NT219 reduced IRS2 expression levels and suppressed viability in a dose-dependent manner (Supplementary Figure S8E and F).

Furthermore, we investigated the impact of NT219 on CRC patient-derived explants (PDE) using the ex vivo CuresponseTM platform. PDEs are particularly effective for rapid, short-term studies and maintain the original tumor microenvironment, unlike patient-derived xenografts. Transcriptomic analysis was performed on CRC PDE-matched samples before and after NT219 treatment. Among the noteworthy pathways significantly enriched in downregulated genes, the most significant was OXPHOS (Supplementary Figure S8G). In accordance, NT219 decreased the expression of OXPHOS genes (Figure 4F), and attenuated basal mitochondrial respiration, ATP production, and maximal respiration in LS513^sh-NS^ cells, while no such effect was observed in LS513^sh-IRS2^ cells (Figure 4G). Taken together, these findings suggest that IRS2 alters the metabolic profile of CRC cells, favoring a shift towards increased reliance on OXPHOS over glycolysis.

### IRS2 Activates β-Catenin in CRC BM

In order to investigate the mechanism of action of IRS2 in the brain environment, we first focused on PI3K/AKT/mTOR pathway, which is closely linked to IRS2.^7^ We examined the effect of HA CM on IRS2-manipulated HCT116 and SW403 cells, using IGF-1 as a positive control and serum-free media (SFM) as a negative control. Enhanced AKT phosphorylation was observed in HCT116^IRS2^ cells within HA CM compared to control cells (Supplementary Figure S9A), and the opposite was seen in SW403^sh-IRS2^ cells (Supplementary Figure S9B). These results suggest that the increased tumorigenic phenotype of IRS2-expressing CRC cells in the brain environment relies, at least partly, on PI3K/AKT activation. Hence, we hypothesized that inhibition of this pathway would be more detrimental to cells overexpressing IRS2. Therefore, we treated cells grown in either HA CM or control media with an AKT1/2 kinase inhibitor (AKTi) (Supplementary Figure S9C and D) or PI3K inhibitor (Alpelisib) (Supplementary Figure S9E and F). Both treatments significantly reduced the proliferation of HCT116^IRS2^ cells compared to the control, but only in the presence of HA CM, where the AKT pathway is activated (Supplementary Figure S9G and I). Similarly, the inhibitory effect of AKTi or Alpelisib on proliferation was more pronounced in SW403^sh-NS^ cells compared to SW403^sh-IRS2^ again in the presence of HA CM (Supplementary Figure S9H and J). Given the relatively subtle effect of AKT inhibition, we hypothesized that additional mechanisms might be involved in mediating IRS2 activity.

**Figure 5.**
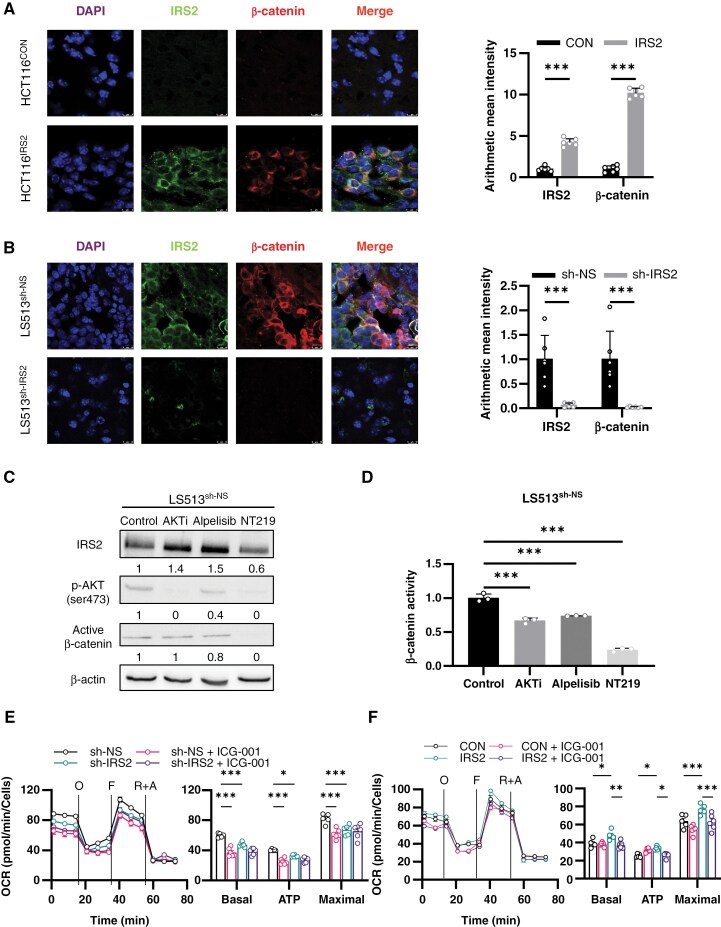
IRS2 rewires β-catenin pathway of CRC cells in the brain microenvironment. (A) Mouse HCT116^CON^ or HCT116^IRS2^ CRC BM tumors described in Figure 2D were immunostained for IRS2 and active β-catenin, scale bars represent 8 µm. Quantification of protein expression was based on representative images from *n* = 3 sections in *n* = 6 mice from each group (two-way ANOVA). (B) Experiment identical to (A) only using mouse LS513^sh-NS^ or LS513^sh-IRS2^ CRC BM tumors described in Figure 2E, scale bars represent 10 µm (two-way ANOVA). (C) LS513^sh-NS^ cells were treated with AKT1/2 kinase inhibitor (AKTi, 1 µM), Alpelisib (1 µM), NT219 (5 µM), or control (D5W). IRS2, pAKT (ser473), and active β-catenin protein levels were determined by western blot and normalized to β-actin level. (D) LS513^sh-NS^ cells were transfected with either pTOPFLASH or pFOPFLASH. A day later, cells were treated as in (C), and luciferase activities were analyzed and normalized to total protein concentration (one-way ANOVA). (E) SW403^sh-NS^ or SW403^sh-IRS2^ were treated with ICG-001 (20 µM) or control vehicle (DMSO) for 4 h. Mitochondrial respiration was measured using Cell Mito Stress Test kit as described in Figure 4D, E; error bars represent s.e.m. (two-way ANOVA). (F) HCT116^CON^ or HCT116^IRS2^ were treated as in (E) and mitochondrial respiration was measured using Cell Mito Stress Test kit as described in Figure 4D, E; error bars represent s.e.m. (two-way ANOVA).

The WNT/β-catenin pathway, a pivotal regulator of CRC metastasis and metabolism,^28^ was another prominent signaling pathway identified in our enrichment analysis (Figure 3B). Further examination illustrated a direct association between IRS2 expression and β-catenin expression and activity (Supplementary Figure S10A and B). Additionally, we demonstrated that IRS2 expression correlated with β-catenin expression in the brain environment by staining the CRC BM tumors shown in Figure 2D and E (Figure 5A and B).

The connection between the PI3K/AKT/mTOR and β-catenin pathways is complex.^29^ In order to study the interaction between IRS2 and β-catenin, we compared inhibition of PI3K/AKT/mTOR pathway to direct inhibition of IRS2 and explored their impact on β-catenin expression and transcriptional activity. Direct inhibition of PI3K or AKT using Alpelisib or AKTi, respectively, had minimal effects on active β-catenin expression, with a 20% reduction or no change (0%) (Figure 5C), and lowered β-catenin transcriptional activity by only 30% (Figure 5D). Similarly, direct inhibition of mTOR with Rapamycin resulted in only a 30% reduction in β-catenin expression (Supplementary Figure S10C). In contrast, NT219 diminished both expression and activity of active β-catenin by 80% (Figure 5C and D). Similar findings were observed in SW403 cells (Supplementary Figure S10C and D). This finding further supports our conclusion that IRS2 functions as an activator of the β-catenin pathway, operating largely independently of the PI3K/AKT/mTOR pathway.

Assuming that the pro-proliferative effect of IRS2 is governed by the β-catenin pathway, blocking β-catenin would likely counteract the impact of IRS2. In order to test this hypothesis, we examined the effects of ICG-001, a β-catenin inhibitor, on the viability of the IRS2-manipulated cells. Indeed, ICG-001 reduced the proliferation of SW403^sh-NS^ cells by 100%, whereas only a 50% reduction was observed in SW403^sh-IRS2^ cells (Supplementary Figure S10E, *P* < 0.0005). A similar trend was observed in the reverse model of HCT116^IRS2^ cells (Supplementary Figure S10F).

Finally, we aimed to evaluate the role of β-catenin in regulating cellular metabolism in IRS2-expressing cells. ICG-001 reduced OXPHOS gene expression in SW403^sh-NS^ (Supplementary Figure S10G) and had a greater impact on the basal mitochondrial respiration, ATP production, and maximal respiration of SW403^sh-NS^ compared to SW403^sh-IRS2^ (Figure 5E). A similar pattern was noted in the reverse model of HCT116^IRS2^ (Figure 5F). These results suggest the involvement of IRS2 in modulating OXPHOS through β-catenin.

### Combination of 5-FU and NT219 Inhibits CRC BM

**Figure 6.**
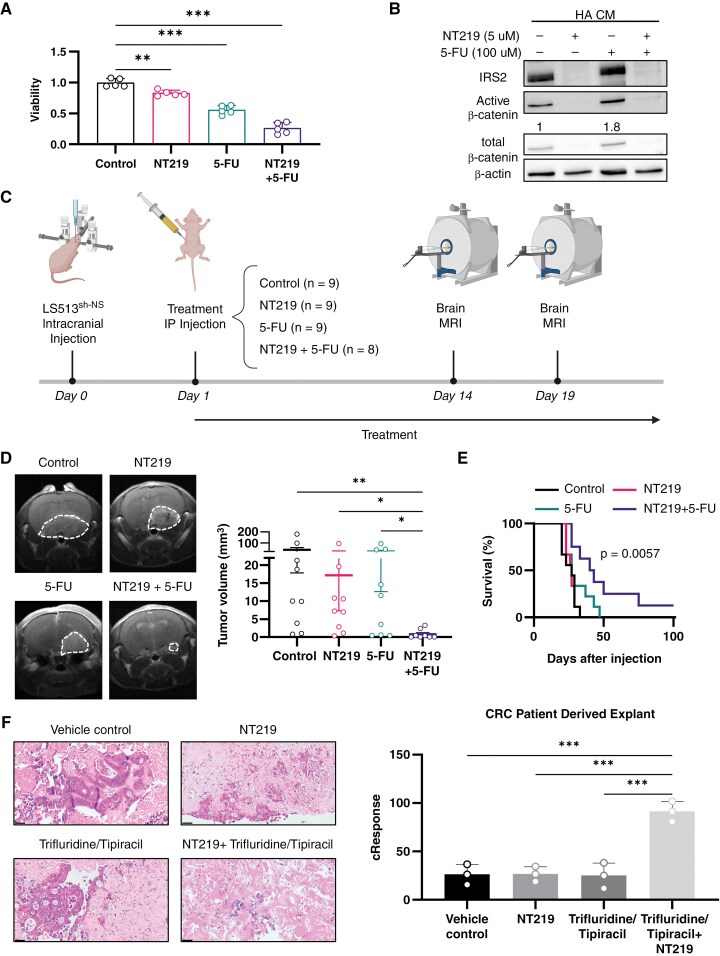
Combination of 5-FU and NT219 inhibits CRC BM and extends animal survival. (A) LS513 cells were treated with either fluorouracil (5-FU) (100 µM) or NT219 (1 µM) or their combination. After 72 h, viability was assessed using methylene blue assay (one-way ANOVA). (B) LS513 cells were seeded, and a day later medium was replaced with HA CM and cells were treated for 24 h with either NT219 (5 µM), 5-FU (100 µM), or their combination. IRS2, pAKT (ser473), and active β-catenin protein levels were determined by western blot and normalized to β-actin level. (C) Experimental design of combined treatment of 5-FU and NT219 in vivo using intracranial LS513^sh-NS^ cells CRC BM mouse model. Illustration was created with BioRender.com. (D) Mice were intracranially injected with LS513^sh-NS^ cells and MRI T1-weighted gadolinium contrast was conducted on d 14 and 19 post cells implantation. Representative MRI image of one mouse brain from each group is shown. Tumor volume was quantified using MRIcro software, error bars represent s.e.m. (Kruskal-Wallis one-way ANOVA). (E) Kaplan–Meyer survival curve of mice was generated (Log-rank (Mantel-Cox) test). (F) Fresh CRC patient-derived biopsy was cultured using the ex vivo platform of *CuresponseTM* and treated with 5% dextrose in water (D5W) control, NT219, Trifluridine/Tipiracil, or a combination of Trifluridine/Tipiracil and NT219. After 96 hours, the samples were fixed, stained with H&E, and blindly evaluated by a pathologist. Analysis using a proprietary AI algorithm measured the functional (cell death) score of response (cResponse). Scale bars represent 50 µm (one-way ANOVA).

We aimed to employ the data accumulated thus far in order to develop a novel treatment strategy for CRC BM. Despite the common use of 5-FU-based therapies for CRC BM, resistance often arises,^3^ partly due to the WNT/β-catenin pathway activation.^30^ Given our data implicating IRS2 in β-catenin activation in CRC cells, we hypothesized that inhibiting IRS2 might enhance CRC cells sensitivity to 5-FU. Indeed, NT219 and 5-FU synergistically decreased LS513 cell proliferation in vitro by 73% (Figure 6A, *P* < 0.001 and Supplementary Figure S11A). Moreover, while 5-FU elevated β-catenin expression within HA CM by 80%, NT219 diminished both 5-FU-induced and basal β-catenin expression levels in LS513 cells (Figure 6B).

We then assessed the combined therapeutic efficacy of 5-FU and NT219 in vivo using our CRC BM mouse model. To this end, LS513 cells were intracranially injected into mice, followed by treatment with 5-FU, NT219, their combination, or a control vehicle a day later (Figure 6C). While neither compound alone had a significant effect, the combination of 5-FU and NT219 significantly and synergistically inhibited tumor growth by 98% (Figure 6D, *P* = 0.006 and Supplementary Figure S11B) and prolonged the median survival by 50% (Figure 6E, *P* = 0.006).

To further explore the effect of NT219 combined with chemotherapy on CRC patient tissues, we utilized the ex vivo platform of CuresponseTM once again. PDE samples were treated with either vehicle control (D5W), NT219, Trifluridine/tipiracil (FTD/TPI), or the combination of NT219 and FTD/TPI. Both FTD/TPI and 5-FU share similar mechanisms of action as antimetabolites interfering with DNA synthesis.^31^ While no response was observed with either NT219 or FTD/TPI alone, a significant and synergistic effect was observed with the combined treatment (Figure 6F). These results suggest that inhibition of IRS2 may sensitize CRC cells to chemotherapy, potentially impacting the management of CRC patients with BM using agents like NT219.

## Discussion

The progress in treating advanced CRC has been substantial, yet CRC BM poses a significant clinical challenge. Understanding the distinct features of CRC BM is crucial for devising effective therapeutic strategies. Our study provides a comprehensive genomic analysis of CRC BM, highlighting the central role of IRS2 in driving their progression. These findings shed light on the underlying mechanisms of CRC BM and offer a potential avenue for targeted intervention.

Identifying GA is a key step in comprehending CRC BM formation. Prior attempts to identify GA in CRC BM were limited by small clinical sample sizes.^18^ Our study stands out for its comprehensive genomic analysis of over 35,000 human CRC primary and metastatic samples, making it the largest analysis of its kind. Using FoundationOne, a targeted capture assay, we observed a significantly increased representation of IRS2 amplification in CRC BM compared to other metastatic sites or primary tumors. Importantly, analysis of clinical samples revealed a higher proportion of CRC BM with elevated IRS2 expression. Thus, while IRS2 amplification initiated this study, heightened IRS2 expression emerges as a more prevalent and clinically significant phenomenon in CRC BM. Additionally, GA may manifest at various stages of cancer development or due to treatment-induced stress.^32^ Our dataset lacked matched local and metastatic samples, preventing differentiation between these conditions.

A potential role of IRS2 in promoting metastasis formation has already been demonstrated in breast cancer^33^ and intrahepatic cholangiocarcinoma,^34^ but its role in CRC has only partially been explored. IRS2 has been suggested as an oncogene in CRC,^9^ and IRS2 copy number gain has been shown to predict the sensitivity of CRC cells to an IGF-1R/IR inhibitor.^21^ Consistent with previous studies, we observed a broad involvement of IRS2 in promoting aggressive behavior among CRC cells. Our study also revealed its specific ability to promote CRC BM formation by employing both in vitro and in vivo models that mimic different organ environments.

To our knowledge, no spontaneous or intracardiac CRC BM mouse models currently exist, unlike the well-established models for melanoma, lung, and breast cancers.^35^ Attempts to establish a US-guided intracardiac model using the murine cell line MC38 have not successfully induced brain parenchymal metastases.^36^ Apart from the intracranial model, the only other CRC BM model described in the literature is the intracarotid injection, which was established using two human cell lines, KM12 and HT29.^37,38^ While intracarotid injection offers a route to simulate hematogenous metastasis, it faces significant technical challenges. In contrast, direct intracranial injection provides a more efficient and accurate approach for establishing BM. However, this model has limitations as it bypasses early metastasis steps such as intravasation, extravasation, and BBB penetration, and may induce neuroinflammation.^35^ Nevertheless, this model mimics the clinical manifestation observed in patients with confirmed substantial metastases, allows for the study of metastatic adaptation to the microenvironment, and is optimal for evaluating new therapeutic strategies for suppressing established lesions.

Our study has revealed that IRS2-expressing CRC cells rely more on OXPHOS than glycolysis. This aligns with findings in melanoma BM, where heightened OXPHOS was observed compared to extracranial metastases. Treatment with IACS-010759, a mitochondrial complex I inhibitor, improved survival and inhibited BM formation in mice resistant to BRAF/MEK inhibitors.^39^ Similar trends were seen in BM from lung, breast, and renal cancers, indicating increased OXPHOS and sensitivity to IACS-010759.^40^ Notably, in our study, many DEGs within the OXPHOS pathway were specifically associated with mitochondrial complex I (Supplementary Figure 7A).

5-FU-based therapies, such as FOLFOX or FOLFIRI, have been used as the standard therapy for CRC BM.^41^ However, BM typically develops at an advanced stage of CRC, accompanied by the emergence of resistance to 5-FU.^3^ Activation of the WNT/β-catenin pathway is one of the main mechanisms responsible for 5-FU resistance.^30^ Notably, the combination therapy involving a WNT inhibitor and 5-FU has effectively inhibited cancer stem cells and reduced CRC tumor recurrence.^42^ Our study showed that a combination of 5-FU and NT219 treatment inhibited the formation of CRC BM and extended animal survival. To our knowledge, this is the first successful preclinical use of a combination of drugs to treat CRC-associated BM. Of note, NT219 as a monotherapy exhibited a lack of in vivo efficacy, despite its effectiveness in vitro. This discrepancy could be due to several factors, including differences in drug pharmacokinetics and pharmacodynamics between in vitro and in vivo systems, especially in terms of BBB permeability, the brain microenvironment, and the presence of other cells and factors that can influence the response to treatment.

Furthermore, high rates of OXPHOS represent another crucial mechanism contributing to 5-FU resistance.^43^ Notably, in patients with 5-FU-exposed CRC resected liver metastases, there was a SIRT1/PGC1α-dependent increase in OXPHOS.^44^ Consistent with these observations, combined treatment with 5-FU and antimycin A, an inhibitor of mitochondrial complex III, has been shown to promote cell death in resistant CRC.^45^ Our study provided molecular insights into how NT219 sensitizes CRC BM to 5-FU by inhibiting β-catenin. However, it is worth noting that part of this sensitization might also be attributed to the suppression of OXPHOS. Further investigation is necessary to fully understand the extent of OXPHOS suppression and its role in 5-FU sensitization in CRC BM.

Taken together, this study identifies the unique genomic profile of CRC BM and highlights the role of IRS2 in promoting CRC BM. These effects may be mediated, at least in part, by modulation of the β-catenin and OXPHOS pathway. Approaching CRC BM may benefit clinically from the inhibition of the IRS pathway with agents such as NT219.

## Supplementary material

Supplementary material is available online at *Neuro-Oncology* (https://academic.oup.com/neuro-oncology).

noaf028_suppl_Supplementary_Table_S1

noaf028_suppl_Supplementary_Table_S2

noaf028_suppl_Supplementary_Material_Table_S7-S11

noaf028_suppl_Supplementary_Table_S3

noaf028_suppl_Supplementary_Table_S4

noaf028_suppl_Supplementary_Table_S5

noaf028_suppl_Supplementary_Table_S6

noaf028_suppl_Supplementary_Figure_S1

noaf028_suppl_Supplementary_Figure_S2

noaf028_suppl_Supplementary_Figure_S3

noaf028_suppl_Supplementary_Figure_S4

noaf028_suppl_Supplementary_Figure_S5

noaf028_suppl_Supplementary_Figure_S6

noaf028_suppl_Supplementary_Table_S12

noaf028_suppl_Supplementary_Table_S13

noaf028_suppl_Supplementary_Table_S14

noaf028_suppl_Supplementary_Figure_S7

noaf028_suppl_Supplementary_Figure_S8

noaf028_suppl_Supplementary_Figure_S9

noaf028_suppl_Supplementary_Figure_S10

noaf028_suppl_Supplementary_Figure_S11

## Data Availability

The sequence data generated in this study have been deposited in the Gene Expression Omnibus (GEO) and are accessible through the GEO Series accession number GSE203017. The remaining data are available within the Article, Supplementary Information, or available from the authors upon request.
