# Supplementary material for: IRS2 as a driver of brain metastasis in colorectal cancer: A potential target for novel therapeutic strategies
Source: Neuro Oncol. 2025 Jan 31;27(7):1729–45. doi: 10.1093/neuonc/noaf028 (PMC12417839; doi:10.1093/neuonc/noaf028)
Supplement: noaf028_suppl_Supplementary_Material_Table_S7-S11 [file noaf028_suppl_supplementary_material_table_s7-s11.docx]

# **Supplementary methods**

**Comprehensive genomic profiling (CGP)**

We assessed all known and likely pathogenic alterations across all classes of GA including short variant, copy number, and rearrangement alterations. Briefly, base substitutions were detected using a Bayesian method allowing the detection of somatic mutations at low MAF with increased sensitivity for mutations at hotspot sites. Indels were identified using a de Bruijn approach. Copy number events were detected by fitting a statistical copy-number model to normalized coverage and allele frequencies at all exons and ~3,500 genome-wide, single-nucleotide polymorphisms. Rearrangements were detected through an analysis of chimeric reads. TMB was determined on 0.8–1.1 Mb as described previously^1^. At the time of the analysis, the dataset consisted of 20,858 local-biopsied and 15,139 distantly metastatic CRC samples.

**CRC Patient-derived Explant (PDE)**

Freshly resected CRC tumors were cut into 250 μm slices using a vibratome (VF300, Precisionary Instruments). A tissue sample was immediately fixed in 4% paraformaldehyde as a reference and analyzed for viability within 24 h. The rest of the slices were placed in 12 or 24 well plates on titanium grids with 4 mL of DMEM/F12 medium, supplemented with 5% FCS, penicillin 100 IU/mL, streptomycin 100 μg/mL, amphotericin B 2.5 μg/mL, gentamicin sulfate 50 mg/mL, and L-glutamine 100 μL/mL. The tissue slices were cultured at 70 rpm on an orbital shaker (TOU-120 N, MRC) at 37°C, 5% CO2. One day after sectioning, CRC sections were treated with D5W, NT219 100 μM, and/or Trifluridine/tipiracil for 96 h where medium and treatment refreshment was performed 48hrs following treatment initiation. At the end of the incubation period, tissue sections were fixed overnight with 4% PFA, followed by FFPE for histology. H&E slides were blindly evaluated by a pathologist to assess the viability of live treated tumor cells on a scale of 0–100% and the level of damage to the tumoral tissue, compared to the immediately fixed and control samples. The analysis included functional (cell death) score of response (cResponse) using proprietary algorithm (Curesponse, Israel) integrating several parameters including pathological evaluation.

**Cells and transfections**

The human primary CRC cell lines were grown in Dulbecco's Modified Eagle's Medium (DMEM) containing 10% fetal bovine serum (FBS). All cells were grown at 37°C in a humidified 5% CO_2_ atmosphere. All transfections used jetPEI (Polyplus-transfection).

**Chemicals**

NT219 (Purple Biotech, Israel); 5-FU (Adooq Bioscience); ICG-001 and Alpelisib (BYL71) (Selleck Chemicals); AKT1/2 kinase inhibitor (AKTi) (Sigma-Aldrich); IGF-1 (PeproTech Inc); Rapamycin (Cayman Chemical).

**Subcutaneous mouse model**

2 × 10^6^ HCT116 cells were injected subcutaneously into six-week-old male athymic nude mice (Envigo CRS, Israel). The size of the tumor was measured every 4-7 days. The formula for estimating tumor volume is V = 1/2 × L × S2 (L: long diameter, S: short diameter). The mice were sacrificed after 29 days and tumors were excised.

**MRI**

MRI was performed with a 7T/30 MR Biospec (Bruker) equipped with a gradient unit of 660mT/m. MRI scans were acquired using a cross coil setup including 86mm resonator and quadrature mouse head coil. Animals’ body temperature was maintained by circulating warm water and their respiration was monitored. Mice were placed in the magnet with the ears positioned at the isocenter. T1-weighted 2D images with rapid acquisition with relaxation enhancement (RARE) sequence were acquired as following: TR/TEeff =800/14.8 ms, RARE factor 4, matrix size 256 × 256, slice thickness 0.5 mm, field of view 1.5 cm, resolution 0.08 × 0.08 mm2, number of averages 12, duration 6min. To enhance the brain with MR contrast, Gd-DTPA (Magnevist, Bayer HealthCare Pharmaceuticals) was administered by IP injection (6.5 mmol/kg) 20 minutes prior to MRI. In cases of opening the BBB a contrast-enhancement on T1-weighted MRI is expected. Tumor volume was quantified using MRIcro software.

**Immunofluorescence**

Mice were anesthetized with an IP injection of ketamine (150 mg/kg) and xylazine (12 mg/kg) and perfused with PBS followed by 4% Paraformaldehyde (PFA). Brains were harvested, and incubated with 4% PFA for 4 h, followed by 0.5 M of sucrose (Sigma-Aldrich) for 1 h, and 1 M sucrose overnight. The brains were then embedded in optimal cutting temperature (OCT) compound (Scigen Scientific) on dry ice and stored at −80 °C. The samples were cut into 10 μm thick sections, placed on X-tra adhesive slides (Leica Biosystems), and stored at − 20 °C. Sections were pre-incubated with a blocking solution containing 0.5% Tween 20 (Sigma-Aldrich), 1% bovine serum albumin (Sigma-Aldrich), and 3% horse serum (Gibco by Life Technologies) for 1 h and then incubated overnight at 4 °C with primary antibodies: rabbit anti-human IRS2 (ab134101; Abcam) and mouse anti-human active β-catenin (05-665; Upstate Biotechnology). The sections were incubated with Alexa Fluor 488 donkey anti-rabbit IgG (Thermo Fisher Scientific, # A-21206, Dilution 1:200) and Alexa Fluor 633 goat anti-mouse IgG (Thermo Fisher Scientific, # A-21052, Dilution 1:200) secondary antibodies for 1 h. Control slides were incubated with the secondary antibody alone. Stained sections were examined and photographed using an LSM 700 confocal microscope (Zeiss). At least three fields of each individual sample were imaged and quantified using ZEN software (Zeiss).

**Immunohistochemistry (IHC)**

3.5 µm sections were mounted on X-tra Adhesive Precleaned Micro Slides (Leica) and processed by an automated immunostainer (VENTANA BenchMARK ULTRA, Ventana Medical System). Automated immunostaining was performed using the I-View DAB detection kit (Ventana Medical System), according to Ventana program. The I-View DAB detection kit utilizes biotinylated secondary antibodies to locate the bound primary antibody, followed by the binding of Streptavidin-HRP (horseradish peroxidase) conjugate. The complex was then visualized with hydrogen peroxidase substrate and 3, 3'- diaminobenzidine (DAB) tetrahydrochloride chromogen. The incubations were performed at a controlled temperature of 37°C. The sections were then counterstained with Gill's hematoxylin, dehydrated, and mounted for microscopic examination. The samples were blindly scored by a pathologist using the immunoreactive score (IRS). IRS gives a range of 0–12 as a product of multiplication between positive cells proportion score (0–4) and staining intensity score (0–3)^2^.

## RNA sequencing (RNA-seq) and Analysis

*RNA-seq.* RNA-seq was conducted at the Tel-Aviv University Genomics Research Unit and Bioinformatics Unit (Tel-Aviv, Israel). For sequencing: briefly, 1000ng of total RNA was fragmented, followed by reverse transcription and second strand cDNA synthesis. The double-strand cDNA was subjected to end repair, A-base addition, adapter ligation, and PCR amplification to create barcoded libraries.

*Analysis.* Adaptors were identified and removed from the raw sequence reads using TagCleaner. Trimmed reads were aligned to the human reference genome UCSC hg19 (Gencode gene annotations) using STAR 2.6.1a. Mapping was followed by transcriptome-wide abundance quantification using Salmon. Pre-alignment and post-alignment quality control (QC) reports were generated using MultiQC.

**Seahorse analysis**

Cells were plated at a density of 1 × 10^4^ cells per well, 10 replicates for each treatment. On the day of analysis, media were changed to Seahorse XF Base Medium supplemented with glutamine (2 mM) for the glycolysis stress test, or pyruvate (1 mM), glutamine (2 mM), and glucose (10 mM) for the mito stress test followed by incubation at 37°C in a non-CO2 incubator for 1 h. Mitochondrial respiration was measured under basal conditions followed by the sequential addition of oligomycin (2 μM), FCCP (0.5 μM), rotenone (0.5 μM), and antimycin A (0.5 μM). Glycolysis activity was measured following sequential addition of glucose (10 mM), oligomycin (1 μM), and 2-deoxy-glucose (2-DG; 50 mM). After each injection, four time points were recorded with approximately 35 minutes between each injection. The OCR and ECAR were automatically recorded and calculated by the Seahorse XF96 Software and normalized to cell number.

**Western blot**

Cells were harvested, lysed, and the total protein was extracted as previously described^3^. Lysates were resolved on 10% SDS-PAGE and immunoblotted with the indicated antibodies: Anti-IRS2 (Abcam; ab134101), Anti-IRS1 (Upstate Biotechnology; 06-248), Anti-Phospho-Akt (Ser473) (Cell Signaling; #9271), Anti-Akt (Cell Signaling; #9272), Anti-active β-catenin (Upstate Biotechnology; 05-665), Anti-β-catenin (BD Transduction; 610154), Anti-Phospho-p70 S6 Kinase (Thr389) (Cell Signaling; #9205), Anti-β-actin (Sigma; A5441).

**Colony assay**

Cells were cultured at low density for two weeks and proceed as previously described^3^.

**Methylene blue assay**

Cells were plated (5 x 10^3^ cells/well) and after 24 h medium was changed to the appropriate media (*e.g.*, regular media or HA CM) or control media (depending on the experiment) and incubated at 37°C, 5% CO_2_ for 24h. Methylene blue assay was performed as previously described^3^ and normalized to viability before treatment.

**Invasion assay**

Cells were plated (5 x 10^4^ cells/insert) into the upper side of Matrigel coated 24 transwell inserts, with pore sizes of 8 µm ([Corning](https://www.google.com/search?rlz=1C1GGRV_enIL752IL752&sxsrf=ALeKk00IP4UUrEXnIYYGoUPpRMRRVm6QEQ:1594115211139&q=Corning+(city),+New+York&stick=H4sIAAAAAAAAAOPgE-LUz9U3MKxMMi5RAjONkguLzbW0spOt9POL0hPzMqsSSzLz81A4VhmpiSmFpYlFJalFxYtYJZzzi_Iy89IVNJIzSyo1dRT8UssVIvOLsnewMgIAA5Rq7WIAAAA&sa=X&ved=2ahUKEwim7o_37brqAhXBepoKHXY_BYYQmxMoATAzegQIChAD)), in media without FBS, whereas the lower chamber contained media with 10% FBS. After 48 h, the upper side of the apical chamber was scraped gently with cotton swabs to remove non-invading cells, and invading cells were fixed and stained with crystal violet.

**Migration (Transwell) assay**

Cells were plated as for invasion assay, only the inserts were not covered with Matrigel.

**3D sphere formation assays**

CRC cells were seeded (2 x 10^3^ cells/drop) in GravityPLUS plates (Insphero) and after four days, spheres were transferred to GravityTRAP plates. Viability of spheres was determined after seven days using Realtime-Glow MT assay (Promega) and spheres were photographed at the end of the experiment.

*CM experiments.* CRC cells were seeded as above. After two days, spheres were transferred to GravityTRAP and medium was changed to HA CM or control media. Viability of spheres was determined after five days using Realtime-Glow MT assay and spheres were photographed at the end of the experiment.

*HA experiments*. HA were seeded in GravityPLUS plates. After three days, spheres were transferred to GravityTRAP plates and IRS2-overexpressed or control empty vector cells expressing m-cherry were added. Cells were photographed and viability assessed by m-cherry quantification using IVIS Lumina III instrument (PerkinElmer) after 24, 48 and 72 h.

**Luciferase assay**

Cells were plated in 12-well plates (1 x 10^5^ cells/well) and transfected with either pTOPFLASH (Upstate Biotechnology; 17-285) or pFOPFLASH (Upstate Biotechnology; 17-285). Forty-eight hours after transfection, luciferase assay was conducted using the Luciferase Assay System kit (Promega) according to the manufacturer's instructions. In all assays, FOPFLASH activity was measured by replacing the pTOPFLASH with pFOPFLASH under equivalent conditions. Luciferase units were normalized to total protein concentration.

**Growth factors analysis**

HA CM secretome was analyzed by Quantibody® Human Growth Factor Array (RayBiotech, Inc.), according to manufacturer's protocol.

## Drug interaction analysis

The model predicts that if the individual drugs have the inhibitory effects *f_1_* and *f_2_* then the expected combined effect of the two drugs is:

*E(f_12_) = 1 – (1 – f_1_) (1 - f_2_) = f_1_ + f_2_ – f_1_ f_2_*

Excess over Bliss (*eob*) is calculated by *eob = f_12_ - E(f_12_)*, where *f_12_* is the observed combined effect. A positive, negative, or null value, is used to determine a synergistic, antagonistic or no interaction, respectively.

## Statistical analysis

All tests were two-tailed. For in vitro experiments, data represent mean and standard deviation (s.d.), and for in vivo experiments, data represent mean and standard error of the mean (s.e.m.). For data with normal distribution, a Student’s t-test/ANOVA (one-way/two-way/repeated measures) was used according to the experimental setup. For data with non-normal distribution, a Kruskal–Wallis test was used. For Kaplan–Meier survival curves, a log-rank test was used. A P value of ≤ 0.05 was considered statistically significant.

**References**

1. Chalmers ZR, Connelly CF, Fabrizio D, et al. Analysis of 100,000 human cancer genomes reveals the landscape of tumor mutational  burden. *Genome Med*. 2017;9(1):34. doi:10.1186/s13073-017-0424-2

2. Fedchenko N, Reifenrath J. Different approaches for interpretation and reporting of immunohistochemistry analysis results in the bone tissue - a review. *Diagn Pathol*. 2014;9:221. doi:10.1186/S13000-014-0221-9

3. Zinger L, Merenbakh-Lamin K, Klein A, et al. Ligand-binding Domain-activating Mutations of ESR1 Rewire Cellular Metabolism of  Breast Cancer Cells. *Clin Cancer Res*. Published online February 2019. doi:10.1158/1078-0432.CCR-18-1505

# **Supplementary Figure legends**

**Supplementary Fig. 1: Genomic divergence between CRC BM, local tumors, and other metastatic sites.**

**A,** CDK8, **B,** FLT3, **C,** ERBB2, and **D,** BCL2L1 alteration in CRC BM compared to local and different metastatic sites (Fisher’s exact test with FDR-corrected p-value), **E,** Distribution of genomic alteration classes, **F,** Distribution of 3,548 CRC clinical samples, analyzed for GA, according to the biopsy site. **G,** The graph depicts the most significantly altered genes in CRC BM compared to Primary. (Fisher’s exact test with FDR-corrected p-value). **H,** IRS2 amplification in CRC BM compared to primary and different metastatic sites (Fisher’s exact test with FDR-corrected p-value).

**Supplementary Fig. 2: IRS2 expression in human CRC samples and cell lines.**

**A,** IRS2 IHC staining of normal colon tissue used as a negative control. Scale bars represent 50 µm. **B,** IRS2 IHC staining of cell blocks of HEK293 cells transfected with pReceiver-Lv247-Empty control (Control) or pReceiver-Lv247-IRS2 (IRS2) negative and positive controls, respectively. Scale bars represent 0.4 mm. **C,** Kaplan-Meier survival curves in brain metastases with low versus high IRS2 expression levels. **D,** Univariate and multivariate analysis for predictors of all-cause mortality. Expression of IRS2. **E,** mRNA (one-way ANOVA) and **F,** protein in HCT116, HT29, SW480, SW403, and LS513 CRC cells determined by qRT-PCR and western blot, respectively, and normalized to β-actin level and HCT116 cells. **G,** HT29 and SW480 cells were infected with pReceiver-Lv247-IRS2 (IRS2) or pReceiver-Lv247-Empty control (CON), and SW403 and LS513 cells were infected with shRNA against IRS2 (sh-IRS2) or nonspecific sequence control (sh-NS). IRS2 protein levels were determined by western blot and normalized to β-actin level.

**Supplementary Fig. 3: IRS2 enhances tumorigenicity of CRC cells.**

**A,** Cells were seeded and after 72 h viability was assessed and normalized to time 0 using methylene blue assay (unpaired t test, two-tailed). **B,** Cells were seeded at low density, and after 15 d, cells were fixed, and colonies were stained and quantified (unpaired t test, two-tailed). **C,D,** Cells were seeded in transwell migration assay **(C)** or transwell invasion assay **(D)** and the number of cells was quantified after 48 h (unpaired t test, two-tailed). **E,** Spheres were generated using inSphero assay, and their viability was evaluated by Realtime-Glow MT after 7 d (unpaired t test, two-tailed).

**Supplementary Fig. 4: IRS2 increases tumorigenicity of CRC cells within the brain environment.**

**A,** Cells were seeded in HA CM or control (HA SFM) using inSphero assay. As those conditions did not support 3D sphere generation, we utilized the assay for 2D culture viability. Viability was evaluated by Realtime-Glow MT (two-way ANOVA). **B,** Spheres were generated, and medium was changed to HA CM or control (HA SFM). Viability was evaluated by Realtime-Glow MT and spheres were photographed after 72 h (two-way ANOVA). **C,** Spheres of HA were generated, and after 3 d, HCT116^CON^ or HCT116^IRS2^ cells expressing m-cherry were added. Cells were photographed after 24, 48, and 72 h. Viability was evaluated at the indicated time by m-cherry quantification using IVIS (repeated measures ANOVA). **D,** Cells were seeded in Microglia CM or control (Microglia SFM) using inSphero assay. As those conditions did not support 3D sphere generation, we utilized the assay for 2D culture viability. Viability was evaluated by Realtime-Glow MT (two-way ANOVA). **E,** Spheres were generated, and medium was changed to Microglia CM or control (Microglia SFM). Viability was evaluated by Realtime-Glow MT and spheres were photographed after 72 h (two-way ANOVA).

**Supplementary Fig. 5: IRS2 specifically enhances the tumorigenic potential of CRC cells within the brain environment.**

**A,** Cells were seeded in Lung CM or control (Lung SFM) using inSphero assay. As those conditions did not support 3D sphere generation, we utilized the assay for 2D culture viability. Viability was evaluated by Realtime-Glow MT (two-way ANOVA). **B,** Spheres were generated, and medium was changed to Lung CM or control (Lung SFM). Viability was evaluated by Realtime-Glow MT and spheres were photographed after 72 h (two-way ANOVA). **C,** Heatmap showing Log_2_ values of growth factors in control (HA SFM) or HA CM measured by a quantitative proteomics array (two-way ANOVA). **D,** HGF, IGFBP-2, insulin, and VEGF expression level in HA, Liver, and Lung cm compared to control (SFM) (two-way ANOVA).

**Supplementary Fig. 6: IRS2 enhances aggressiveness-associated gene signatures in CRC cells.**

Validation of chosen downregulated genes related to metastasis formation in **(A)** SW403^sh-IRS2-2^ compared to SW403^sh-NS^ cells and **(B)** HCT116^IRS2^ compared to HCT116^CON^ cells by qRT-PCR (two-way ANOVA). **C-E,** same as in **(A,B)** under HA CM conditions.

Transcriptomic analysis of HCT116^CON^ or HCT116^IRS2^ cells grown under standard conditions was performed using RNAseq. **F,** Volcano plot of differential gene expression between HCT116^CON^ and HCT116^IRS2^ cells. Each circle represents a gene. Red color refers to significantly upregulated genes (p-value<0.05, fold change≥2), while blue color refers to significantly downregulated genes (p-value<0.05, fold change≤0.5). **G,** Barplot visualization of all significantly over-represented MSigDB curated “Hallmark” and “Canonical KEGG” gene sets in HCT116^CON^ versus HCT116^IRS2^. The color of the bars represents the P value for each enriched gene set identified by Fisher’s exact test. The bar length represents the number of differentially expressed genes enriched in each gene set.

**Supplementary Fig. 7: IRS2 enhances mitochondrial activity.**

**A,** KEGG pathway illustration of OXPHOS in human. Significantly down-regulated genes are labeled by red. No up-regulated genes met the criteria for statistical significance.

Validation of chosen down-regulated genes related to OXPHOS by qRT-PCR in **(B)** SW403^sh-NS^ compared to SW403^sh-IRS2^, **(C)** SW403^sh-NS^ compared to SW403^sh-IRS2-2^,**(D)** LS513^sh-NS^ compared to LS513^sh-IRS2^, and **(E)** HCT116^CON^ compared to HCT116^IRS2^ (unpaired t test, two-tailed).

**Supplementary Fig. 8: IRS2 functions independently of glycolysis.**

**A,B** Glycolytic activity was studied by monitoring extracellular acidification rate (ECAR) using Seahorse Glycolysis Stress Test Kit. **A,** HCT116^CON^ or HCT116^IRS2^ or **(B)** SW403^sh-IRS2^ or SW403^sh-NS^ were seeded and ECAR was measured following sequential addition of glucose (G), oligomycin (O), and 2-DG. Bar chart showing glycolysis, glycolytic capacity, and glycolytic reserve; Error bars represent s.e.m. (two-way ANOVA). **C,** HCT116^CON^ or HCT116^IRS2^ or **(D)** SW403^sh-IRS2^ or SW403^sh-NS^ were treated with 2-DG (20 mM) and after 72 h, viability was assessed using methylene blue assay (two-way ANOVA). **E,** LS513 cells were treated with elevated NT219 concentrations and IRS2 protein levels were determined by western blot and normalized to β-actin level. **F,** LS513 cells were treated with elevated NT219 concentrations and after 72 h, viability was assessed and normalized to time 0 using methylene blue assay (one-way ANOVA). **G,** Fresh CRC patient-derived biopsy was cultured using the ex-vivo platform of *CuresponseTM* and treated with NT219 for 96hr. Transcriptomic analysis was performed on matched samples before and after NT219 treatment. Significantly enriched KEGG pathways, identified using Benjamini correction <0.05, are displayed. Of note, only down-regulated pathways showed significant enrichment.

**Supplementary Fig. 9: IRS2 activates AKT pathway in CRC cells in the brain microenvironment.**

**A,** HCT116^CON^ or HCT116^IRS2^ or **(B)** SW403^sh-NS^ or SW403^sh-IRS2^ were starved for 24 h in serum-free medium (SFM) and then stimulated with SFM or IGF-1 (100 ng/ul) or HA CM for 15 min. IRS2, IRS1, pAKT (ser473), and tAKT protein levels were determined by western blot and normalized to β-actin level. **C,** HCT116^CON^ or HCT116^IRS2^ or **(D)** SW403^sh-NS^ or SW403^sh-IRS2^ were treated with HA CM or SFM (control media) containing AKT1/2 kinase inhibitor (AKTi, 1,000 nM) or control (without AKTi). IRS2, pAKT (ser473), and tAKT protein levels were determined by western blot and normalized to β-actin level. **E,F,** Experiment identical to **(C,D)**, only using Alpelisib. **G,** HCT116^CON^ or HCT116^IRS2^ or **(H)** SW403^sh-NS^ or SW403^sh-IRS2^ were treated with HA CM or control (HA SFM) containing AKTi (500 nM or 1,000 nM) or control (without AKTi). Viability was assessed 72 h later, each group relative to control (HA SFM) and without AKTi, using methylene blue assay (two-way ANOVA). **I,J,** Experiment identical to **(G,H)**, only using Alpelisib (two-way ANOVA).

**Supplementary Fig. 10: IRS2 activates β-catenin pathway in CRC cells in the brain microenvironment.**

**A,** Cells were transfected with either pTOPFLASH or pFOPFLASH. Luciferase activities were analyzed and normalized to total protein concentration (unpaired t test, two-tailed). **B,** IRS2 and active β-catenin protein levels were determined by western blot and normalized to β-actin level. **C,** SW403^sh-NS^ cells were treated with AKT1/2 kinase inhibitor (AKTi, 1 µM), Alpelisib (1 µM), NT219 (5 μM or 10 μM ), Rapamycin (100nM) or control (D5W). IRS2, pAKT (ser473), pp70S6K (Thr389) and active β-catenin protein levels were determined by western blot and normalized to β-actin level. **D,** SW403^sh-NS^ cells were transfected with either pTOPFLASH or pFOPFLASH. A day later cells were treated as in **(C)**, and luciferase activities were analyzed and normalized to total protein concentration (one-way ANOVA). **E,** SW403^sh-NS^ or SW403^sh-IRS2^ or **(F)** HCT116^CON^ or HCT116^IRS2^ cells were treated with elevated ICG-001 concentrations. After 72 h, viability was assessed and normalized to time 0 using methylene blue assay (two-way ANOVA). **G,** SW403 cells were treated with ICG-001 (20 µM) or control vehicle (DMSO). Expression of genes related to OXPHOS was determined by qRT-PCR (two-way ANOVA).

**Supplementary Fig. 11: Combination of 5-FU and NT219 works in synergy *in vitro* and *in vivo.***

**A,B,** Bliss score was assessed for the *in vitro* (described in **Fig. 6A**) and *in vivo* (described in **Fig. 6D**) studies using the formula (Ea + Eb - Ea x Eb), Ea = fractional inhibition obtained by NT219, Eb = fractional inhibition obtained by 5-FU. Excess over Bliss (*eob*) is calculated by the observed combined effect compared with the Bliss score. A positive, negative, or null value, is used to determine a synergistic, antagonistic, or no interaction, respectively.

# **Supplementary Tables**

**Supplementary Table 1: Gene lists for Version 3 (v3) of the FoundationOne Assay** - Excel file

**Supplementary Table 2: Gene lists for Version 5 (v5) of the FoundationOne Assay** - Excel file

**Supplementary Table 3: Genes differentially expressed between silenced and control cells** - Excel file

**Supplementary Table 4: All significantly enriched ORA analysis pathways** - Excel file

**Supplementary Table 5: All significantly enriched GSEA analysis pathways** - Excel file

**Supplementary Table 6: All significant metabolic pathways** - Excel file

**Supplementary Table 7: qPCR Primers list.**

| β-actin (Human) | Forward: GCTCAGGAGGAGCAATGATCTT  Reverse: TTGCCGACAGGATGCAGAA |
| --- | --- |
| IRS2 (Human) | Forward: CCACCATCGTGAAAGAGTGAAGA  Reverse: GCCTTGTTGGTGCCTCATCT |
| DDR2 (Human) | Forward: CCACTATGCAGAGGCTGACA  Reverse: CAGAGATGAACCTCCCCAAA |
| FLT4 (Human) | Forward: GCCATGTACAAGTGTGTGGTCTC  Reverse: ACTTGTAGCTGTCGGCTTGG |
| C-KIT (Human) | Forward: CCGGTCGATTCTAAGTTCTAC  Reverse: GATTGGTGCTCTCTGAAATCTG |
| FGF2 (Human) | Forward: CAAAAACGGGGGCTTCTTCCTG  Reverse: CCATCTTCCTTCATAGCCAGGTAACG |
| CCND2 (Human) | Forward: TCCTGGCCTCCAAACTCAAA  Reverse: AAGTCATGAGGAGTGACAGC |
| LIFR (Human) | Forward: TGTATGTGGTGACAAAGGAAAA  Reverse: TGGATTTGGAATATCAGGGTAGA |
| FOXA1 (Human) | Forward: AGGAACTGTGAAGATGGAAGG  Reverse: ATGTTGCCGCTCGTAGTC |
| NOTCH3 (Human) | Forward: CAAGGGTGAGAGCCTGATGG  Reverse: GAGTCCACTGACGGCAATCC |
| NDUFC2 (Human) | Forward: GGCTTGTCTACATCGGCTTC  Reverse: TGATGGTCCCTCACAGCATA |
| NDUFB8 (Human) | Forward: GCTCCCTGACCGCTCACAGC  Reverse: TGCCAGTGCATCGGTTCACCC |
| NDUFA8 (Human) | Forward: TGTCGCAAACAGCAGGCAAA  Reverse: CTGGGATCCGGTCTTGGTCT |
| COX15 (Human) | Forward: TGGTGTTCCTTACGGCCCTC  Reverse: CCCAGAATCCGGTGATCAAACT |
| NDUFA12 (Human) | Forward: ACATTCTGGGATGTGGATGG  Reverse: CTAGTGGTAGAATAAGGTAC |
| NDUFB10 (Human) | Forward: TAGAGCGGCAGCACGCAAAG  Reverse: CTGACAGGCTTTGAGCCGATC |
| NDUFA11 (Human) | Forward: AAAGCCTACAGCACCACCAG  Reverse: TGTCCAACCTTAGCCACTCC |
| COX11 (Human) | Forward: CTACGCTGCCGTACCCCTTT  Reverse: TGACCTGCAACTGCTGATCCT |
| UQCRC2 (Human) | Forward: AAAGTTGCCCCGAAGGTTAAA  Reverse: GAGCATAGTTTTCCAGAGAAGCA |

**Supplementary Table 8: Demographic properties of Foundation Medicine CRC population.**

|  | **Brain met** | **Other met** | **P value** |
| --- | --- | --- | --- |
| **Median Age** | 60 | 60 | 0.16 |
| **Median TMB** | 5.00 | 3.75 | 2.87E-11 |
| **Fraction Male (%)** | 57.97 | 53.43 | 0.14 |
| **Fraction EUR (%)** | 77.34 | 75.08 | 0.44 |
| **Fraction AFR (%)** | 10.79 | 11.10 | 1 |
| **Fraction EAS (%)** | 1.80 | 3.70 | 0.11 |
| **Fraction SAS (%)** | 0.72 | 0.79 | 1 |
| **Fraction AMR (%)** | 9.35 | 9.34 | 1 |

*AFR* African, *AMR* Admixed American, *EAS* East Asian, *EUR* European, *Met* metastasis, *SAS* South Asian, *TMB* tumor mutational burden.

**Supplementary Table 9: IRS2 amplification in BM compared to local in different cancer types.**

|  | **BM** | **BM + IRS2 amp** | **% BM + IRS2 amp** | **Local** | **Local + IRS2 amp** | **% Local + IRS2 amp** | **OR** | **corrected p value** |
| --- | --- | --- | --- | --- | --- | --- | --- | --- |
| **NSCLC** | 3224 | 26 | 0.8% | 33625 | 215 | 0.6% | 1.3 | 0.404 |
| **Breast** | 781 | 20 | 2.6% | 11277 | 171 | 1.5% | 1.7 | 0.144 |
| **Melanoma** | 538 | 1 | 0.2% | 3002 | 8 | 0.3% | 0.7 | 1 |
| **CRC** | 278 | 21 | 7.6% | 20858 | 598 | 2.9% | 2.8 | 0.001 |
| **Esophagus** | 157 | 3 | 1.9% | 6210 | 46 | 0.7% | 2.6 | 0.319 |
| **Kidney** | 132 | 0 | 0.0% | 2733 | 11 | 0.4% | 0.0 | 1 |
| **Ovary** | 116 | 0 | 0.0% | 5815 | 34 | 0.6% | 0.0 | 1 |
| **Small cell** | 100 | 0 | 0.0% | 1325 | 36 | 2.7% | 0.0 | 0.345 |

*Amp amplification, CRC colorectal cancer*, *Met* metastasis, *NSCLC* Non-small cell lung cancer.

**Supplementary Table 10: IRS2 Amplification and Routine Biomarkers Correlation Analysis**

| **Local tumors** | |  |  |  |  |  |
| --- | --- | --- | --- | --- | --- | --- |
|  | **IRS2(+) Gene(-)** | **IRS2(+) Gene(+)** | **IRS2(-) Gene(-)** | **IRS2(-) Gene(+)** | **OR** | **P value** |
| **KRAS** | 286 | 312 | 10174 | 10086 | 1.1 | 0.26 |
| **NRAS** | 558 | 40 | 19462 | 798 | 1.75 | 0.002 |
| **BRAF** | 531 | 67 | 18002 | 2258 | 1.01 | 0.95 |
| **ERBB2** | 573 | 25 | 19260 | 1000 | 0.84 | 0.44 |
| **APC** | 84 | 514 | 4628 | 15632 | 1.81 | 1.27E-07 |
| **TP53** | 43 | 555 | 5191 | 15069 | 4.45 | 1.34E-30 |
|  |  |  |  |  |  |  |
| **Brain mets** | |  |  |  |  |  |
|  | **IRS2(+) Gene(-)** | **IRS2(+) Gene(+)** | **IRS2(-) Gene(-)** | **IRS2(-) Gene(+)** | **OR** | **P value** |
| **KRAS** | 9 | 12 | 95 | 162 | 0.78 | 0.64 |
| **NRAS** | 16 | 5 | 242 | 15 | 5.04 | 0.01 |
| **BRAF** | 19 | 2 | 233 | 24 | 1.02 | 1 |
| **ERBB2** | 20 | 1 | 233 | 24 | 0.49 | 0.7 |
| **APC** | 3 | 18 | 39 | 218 | 1.07 | 1 |
| **TP53** | 1 | 20 | 41 | 216 | 3.8 | 0.22 |
|  |  |  |  |  |  |  |
| **Other mets** | |  |  |  |  |  |
|  | **IRS2(+) Gene(-)** | **IRS2(+) Gene(+)** | **IRS2(-) Gene(-)** | **IRS2(-) Gene(+)** | **OR** | **P value** |
| **KRAS** | 237 | 317 | 7146 | 7161 | 1.33 | 0.00099 |
| **NRAS** | 511 | 43 | 13665 | 642 | 1.79 | 0.0009 |
| **BRAF** | 515 | 39 | 13345 | 962 | 1.05 | 0.73 |
| **ERBB2** | 538 | 16 | 13578 | 729 | 0.55 | 0.017 |
| **APC** | 58 | 496 | 2661 | 11646 | 1.95 | 3.00E-07 |
| **TP53** | 35 | 519 | 3179 | 11128 | 4.24 | 1.00E-23 |

**Supplementary Table 11: Clinical Characteristics of CRC BM and LM.**

|  |  | **LM** | **BM** | **P value** |
| --- | --- | --- | --- | --- |
| **Median age (range)** |  | 65 (34,88) | 66 (35, 85) | 0.9528 |
| **Sex - no (%)** |  | | | >0.9999 |
|  | Male | 28 (57) | 32 (57) |  |
|  | Female | 21 (43) | 24 (43) |  |
| **Primary** |  | | | 0.6092 |
|  | Right | 17 (35) | 9 (16) |  |
|  | Left | 15 (31) | 13 (23) |  |
|  | Rectum | 13 (27) | 17 (30) |  |
|  | NA | 4 (8) | 27 (48) |  |
| **Brain lesion location (Left, Right, NA)** |  | | | |
|  | Cerebellum |  | 15 (6,4,5) |  |
|  | Frontal |  | 14 (5,7,2) |  |
|  | Parietal |  | 5 (1,4,0) |  |
|  | Occipital |  | 3 (2,1,0) |  |
|  | Temporal |  | 2 (0,2,0) |  |
|  | NA |  | 17 |  |
| **Number of BM** |  | | | |
|  | 1 |  | 42 (75) |  |
|  | 2 |  | 8 (14) |  |
|  | 3 |  | 3 (5) |  |
|  | 4 |  | 1 (2) |  |
|  | 5 |  | 2 (4) |  |
| **BM Symptomatic** |  |  | 56 (100) |  |

Patient and disease characteristics for a cohort of patients with CRC BM and LM.

**Supplementary Table 12: HCT 116 - Genes differentially expressed between overexpression and control cells** - Excel file

**Supplementary Table 13: HCT116 - All significantly enriched ORA analysis pathways** - Excel file

**Supplementary Table 14: HCT116 - All significant metabolic pathways** - Excel file
