# Supplementary figures and images for: IRS2 as a driver of brain metastasis in colorectal cancer: A potential target for novel therapeutic strategies
Source: Neuro Oncol. 2025 Jan 31;27(7):1729–45. doi: 10.1093/neuonc/noaf028 (PMC12417839; doi:10.1093/neuonc/noaf028)

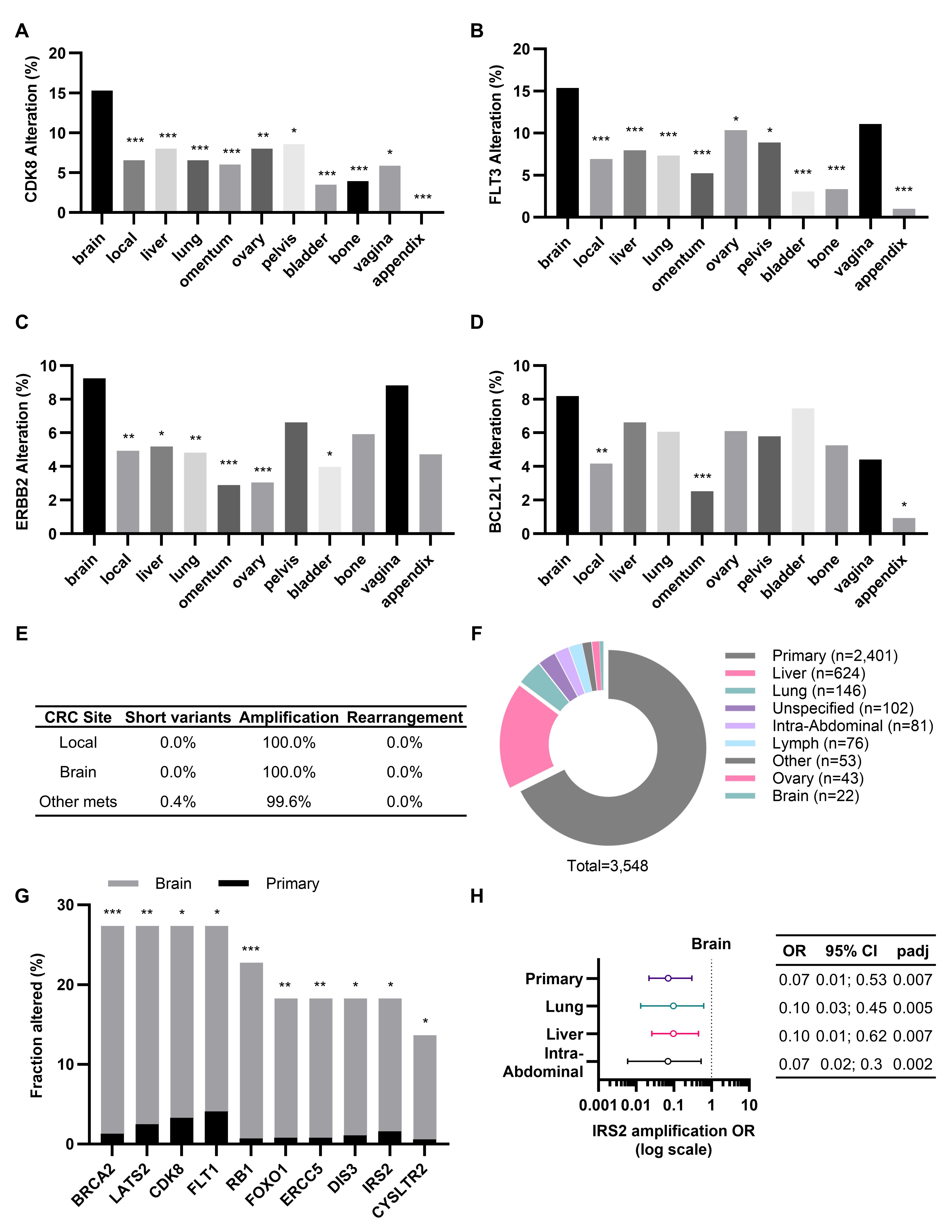

Supplement: noaf028_suppl_Supplementary_Figure_S1 [file noaf028_suppl_supplementary_figure_s1.jpeg]

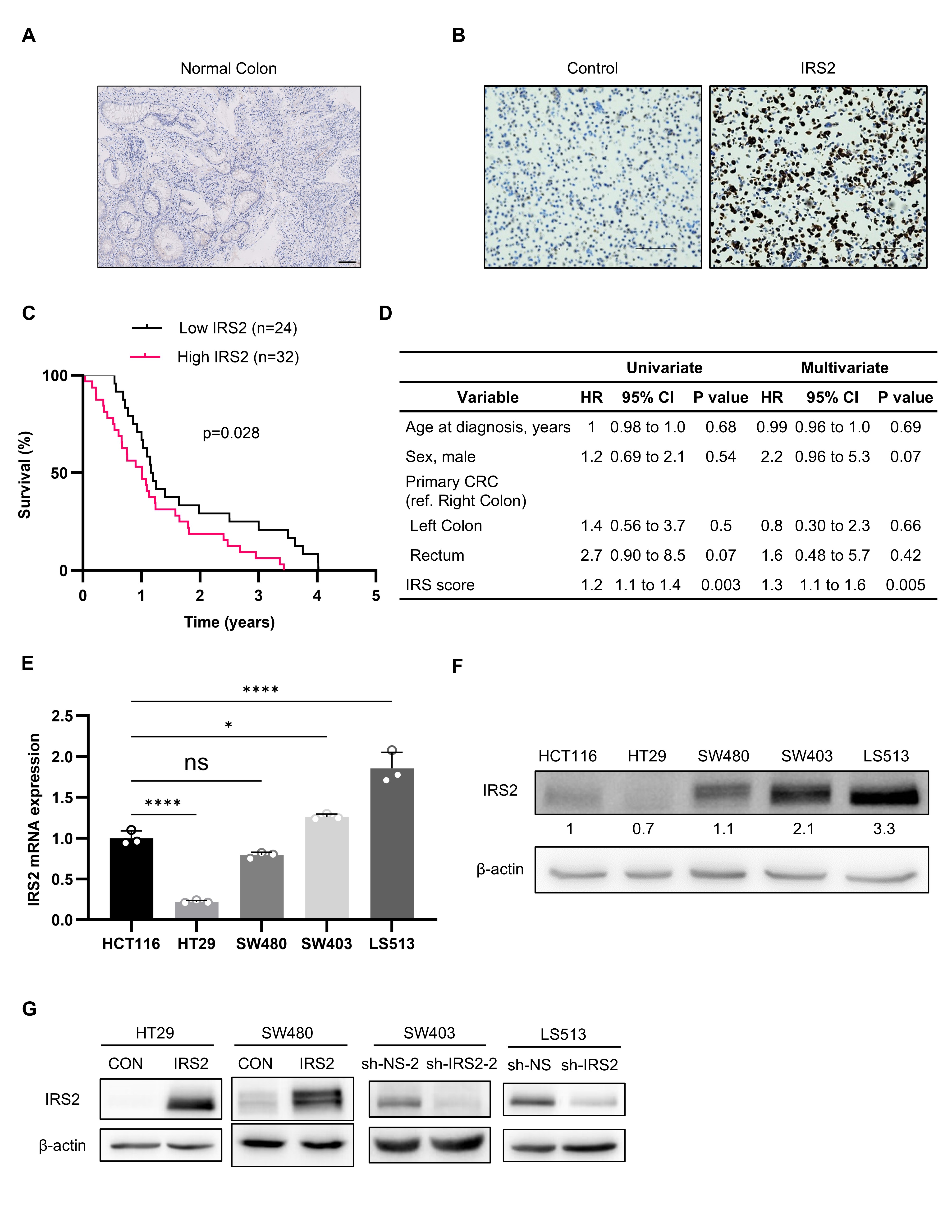

Supplement: noaf028_suppl_Supplementary_Figure_S2 [file noaf028_suppl_supplementary_figure_s2.jpeg]

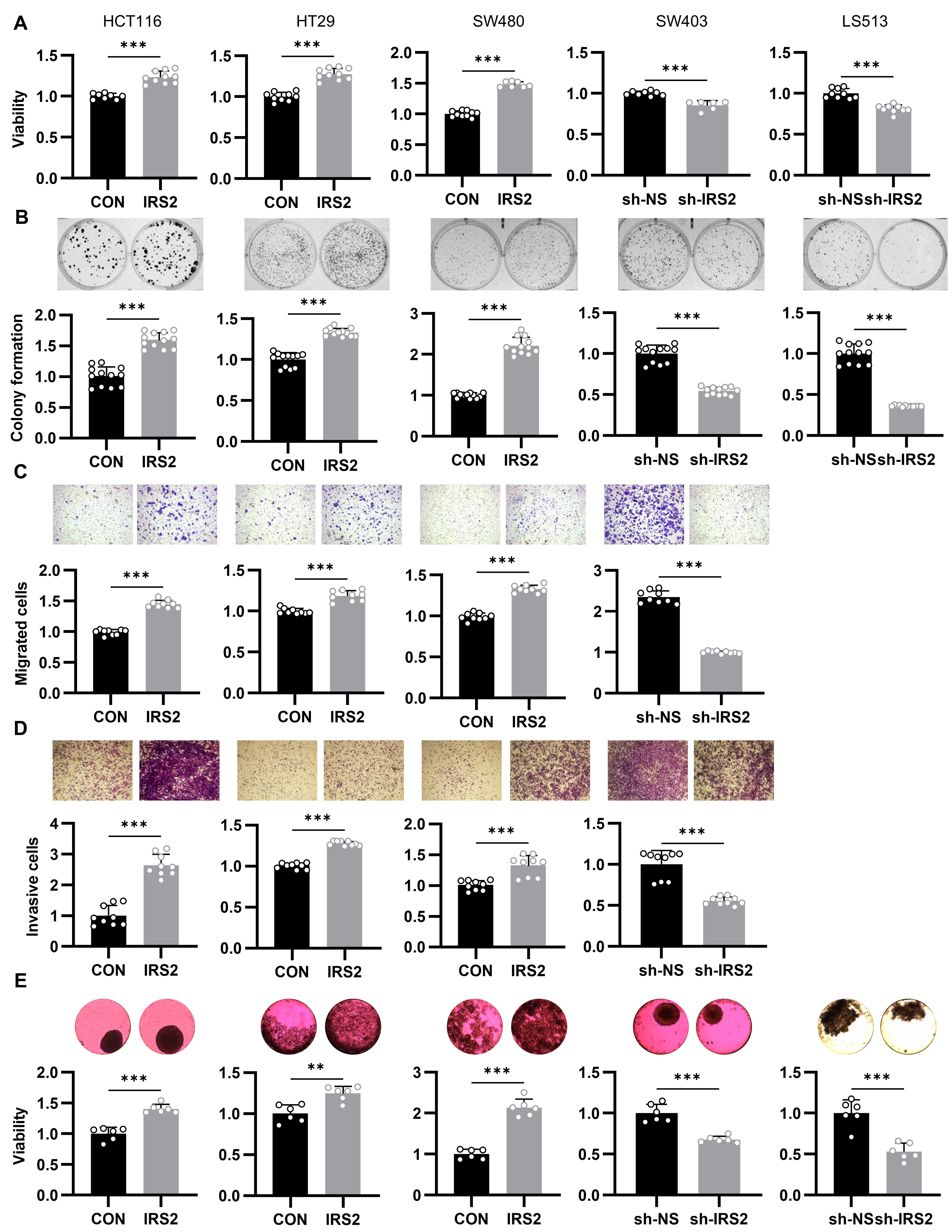

Supplement: noaf028_suppl_Supplementary_Figure_S3 [file noaf028_suppl_supplementary_figure_s3.jpeg]

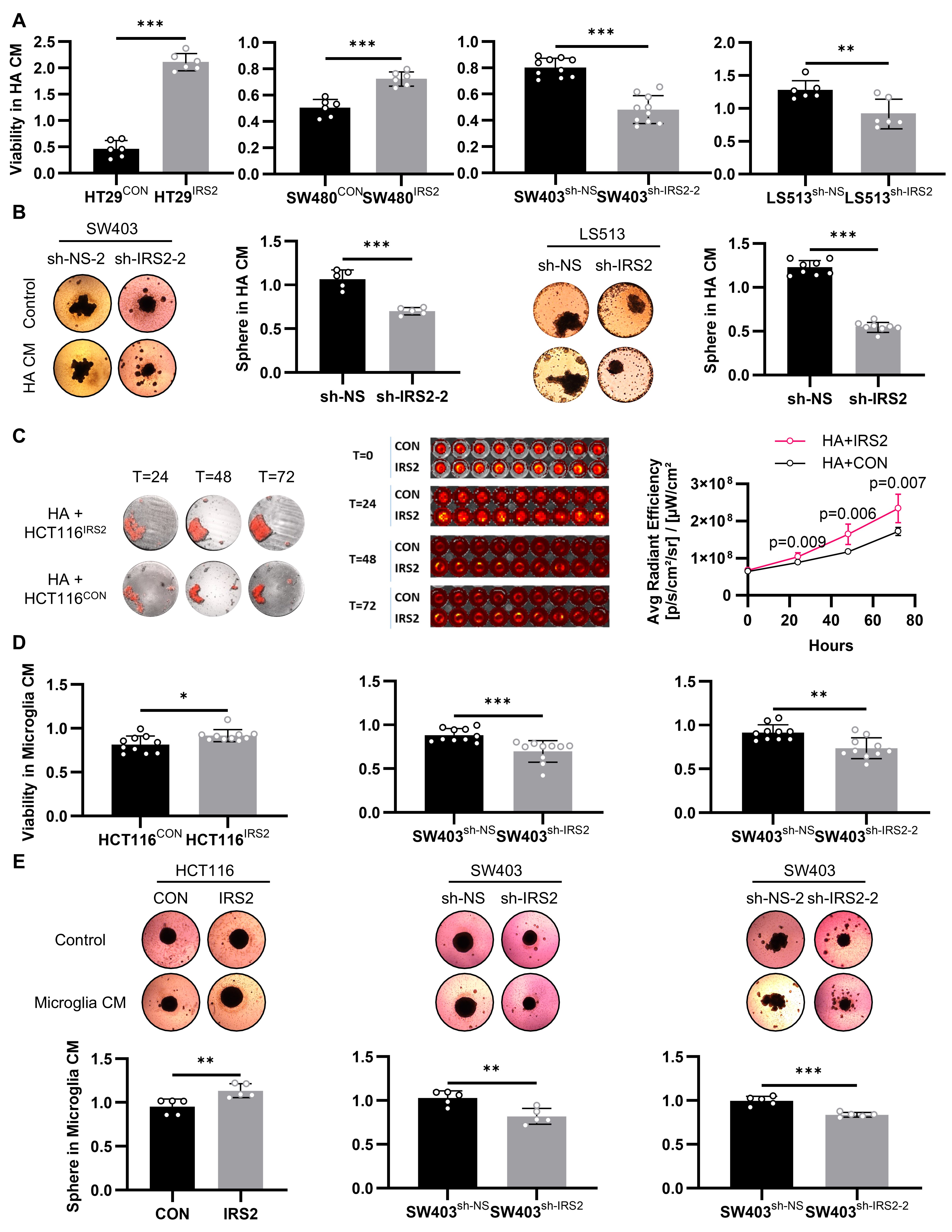

Supplement: noaf028_suppl_Supplementary_Figure_S4 [file noaf028_suppl_supplementary_figure_s4.jpeg]

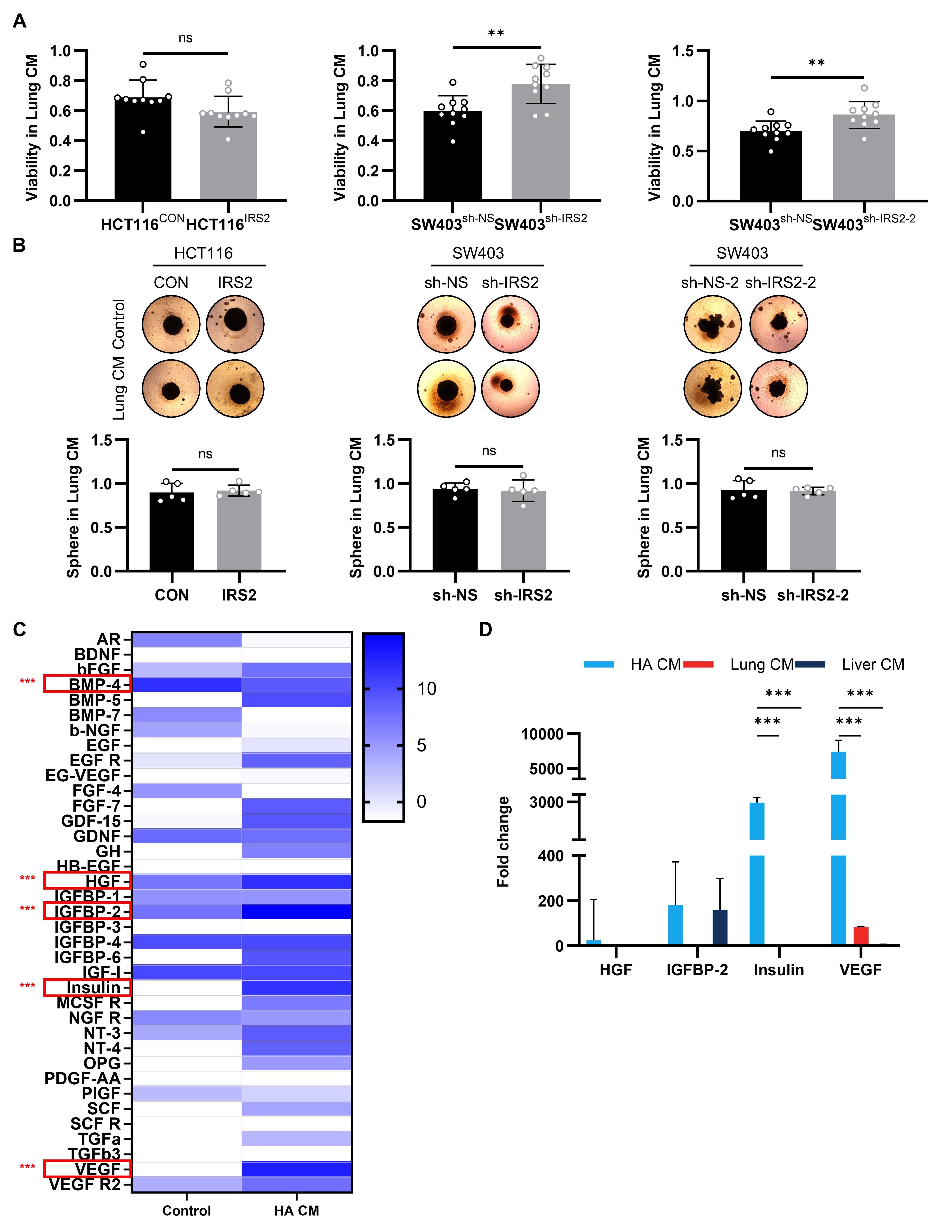

Supplement: noaf028_suppl_Supplementary_Figure_S5 [file noaf028_suppl_supplementary_figure_s5.jpeg]

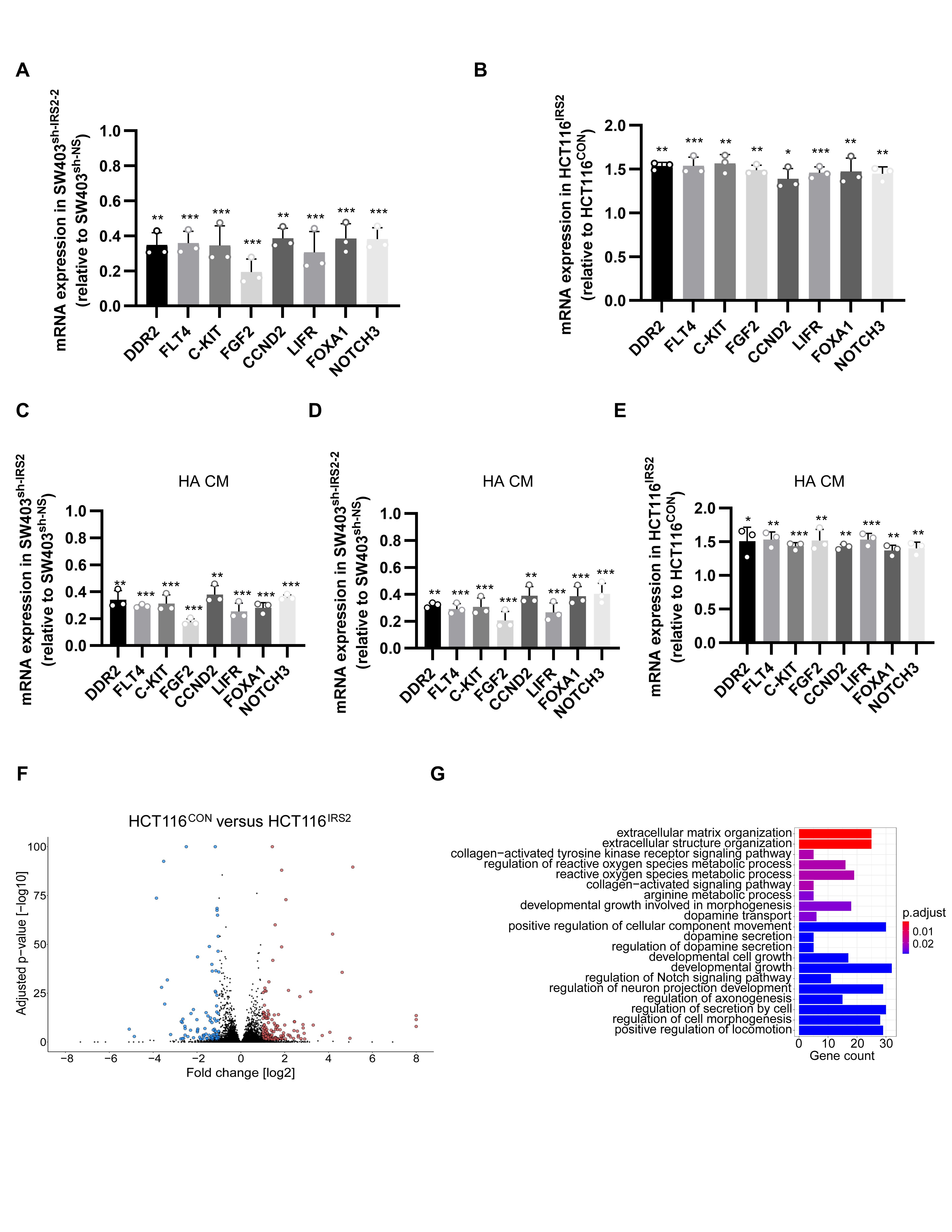

Supplement: noaf028_suppl_Supplementary_Figure_S6 [file noaf028_suppl_supplementary_figure_s6.jpeg]

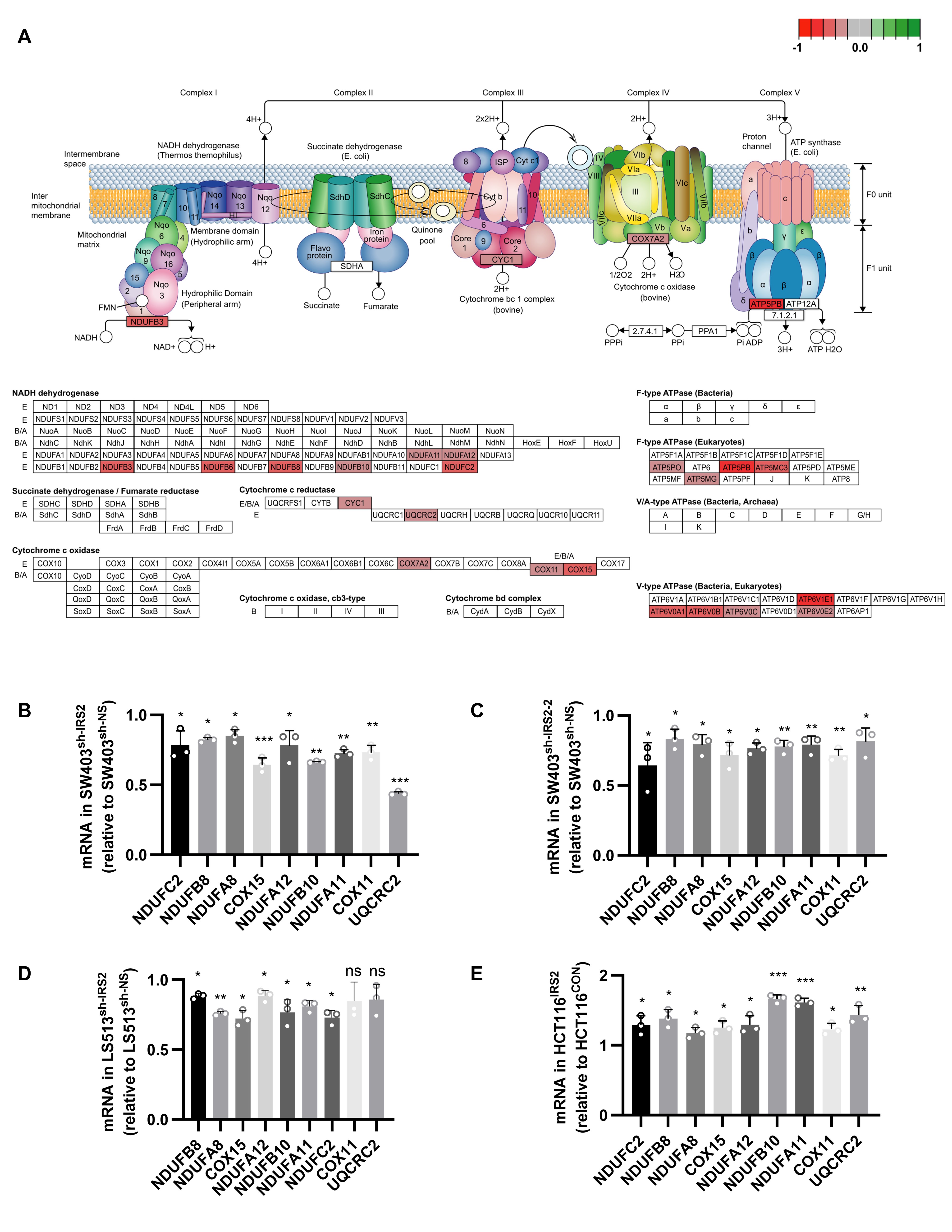

Supplement: noaf028_suppl_Supplementary_Figure_S7 [file noaf028_suppl_supplementary_figure_s7.jpeg]

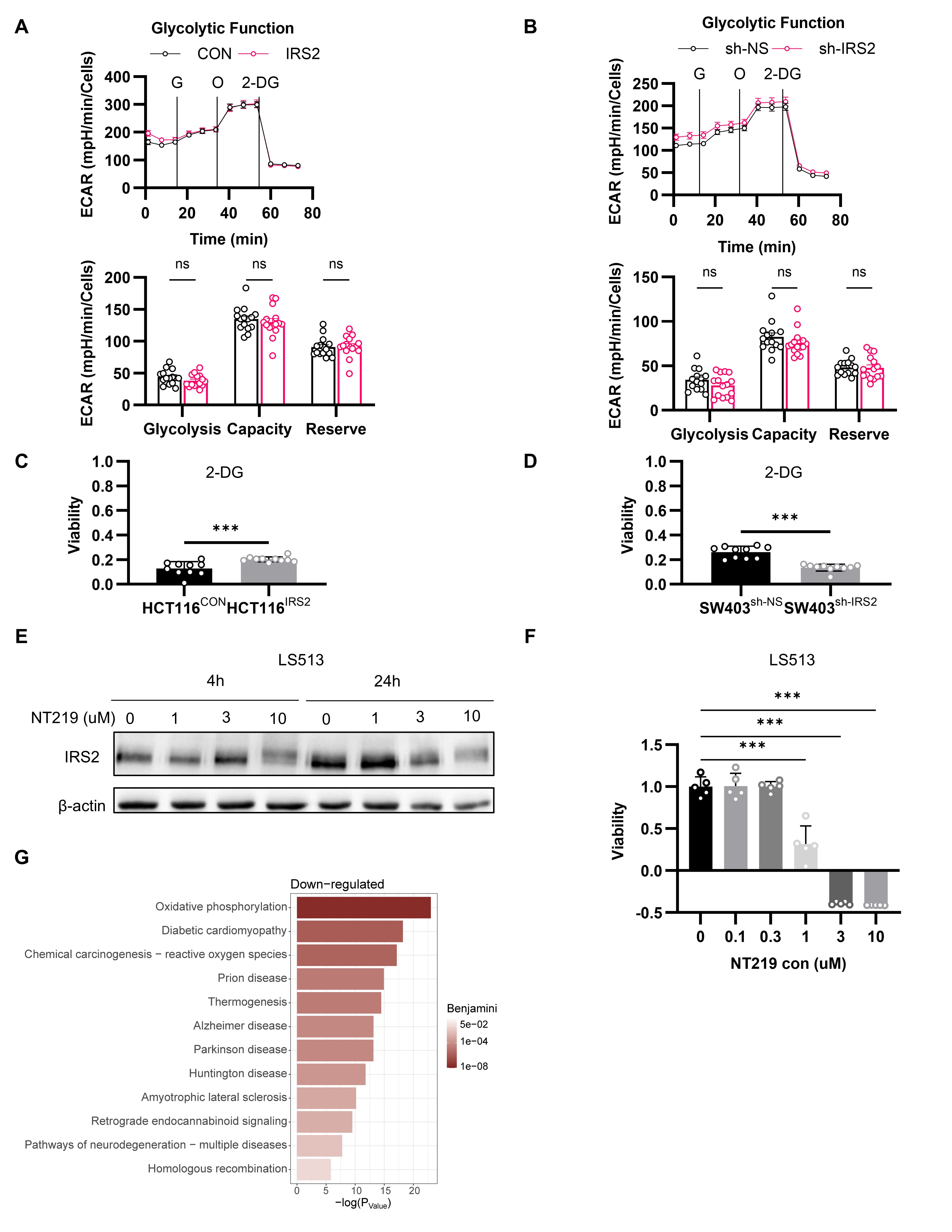

Supplement: noaf028_suppl_Supplementary_Figure_S8 [file noaf028_suppl_supplementary_figure_s8.jpeg]

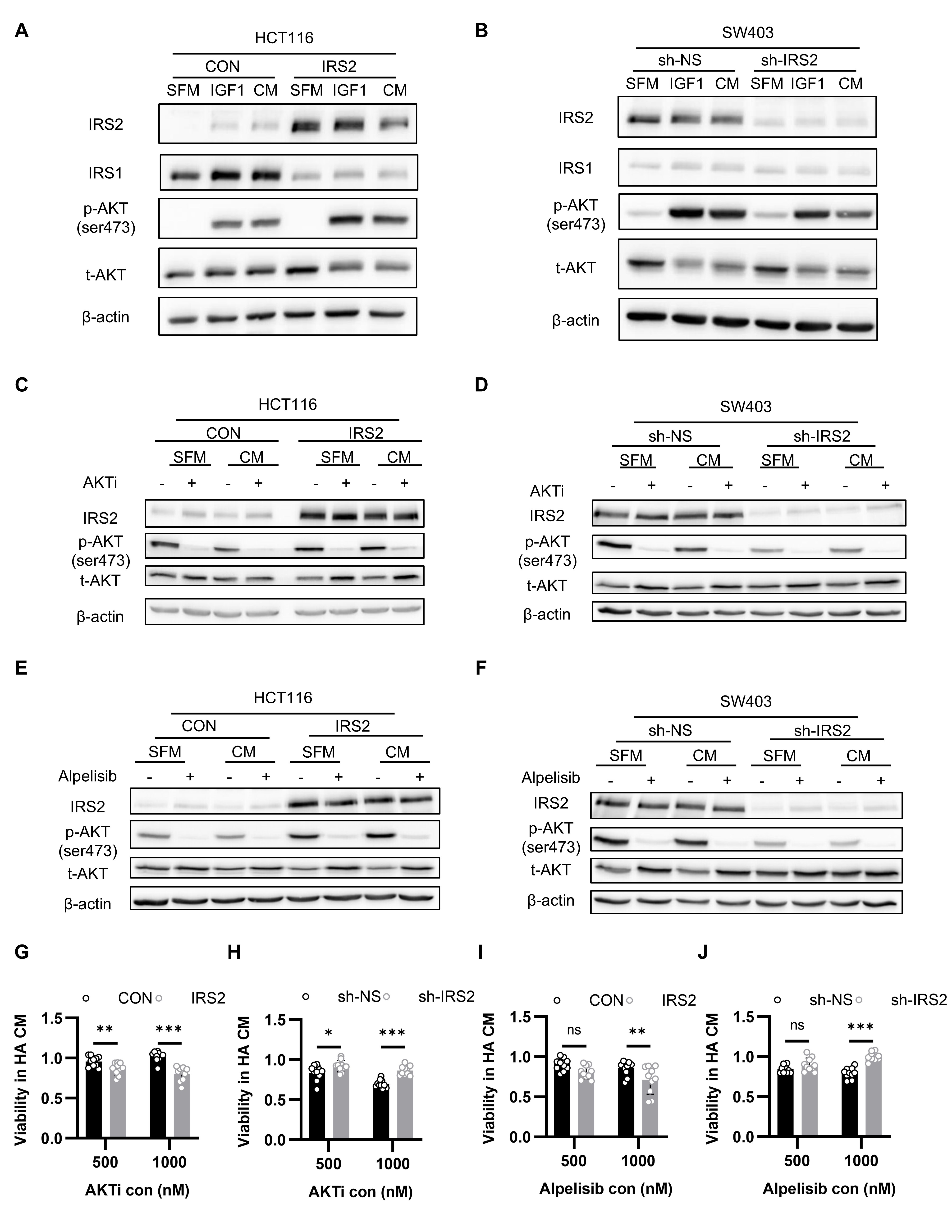

Supplement: noaf028_suppl_Supplementary_Figure_S9 [file noaf028_suppl_supplementary_figure_s9.jpeg]

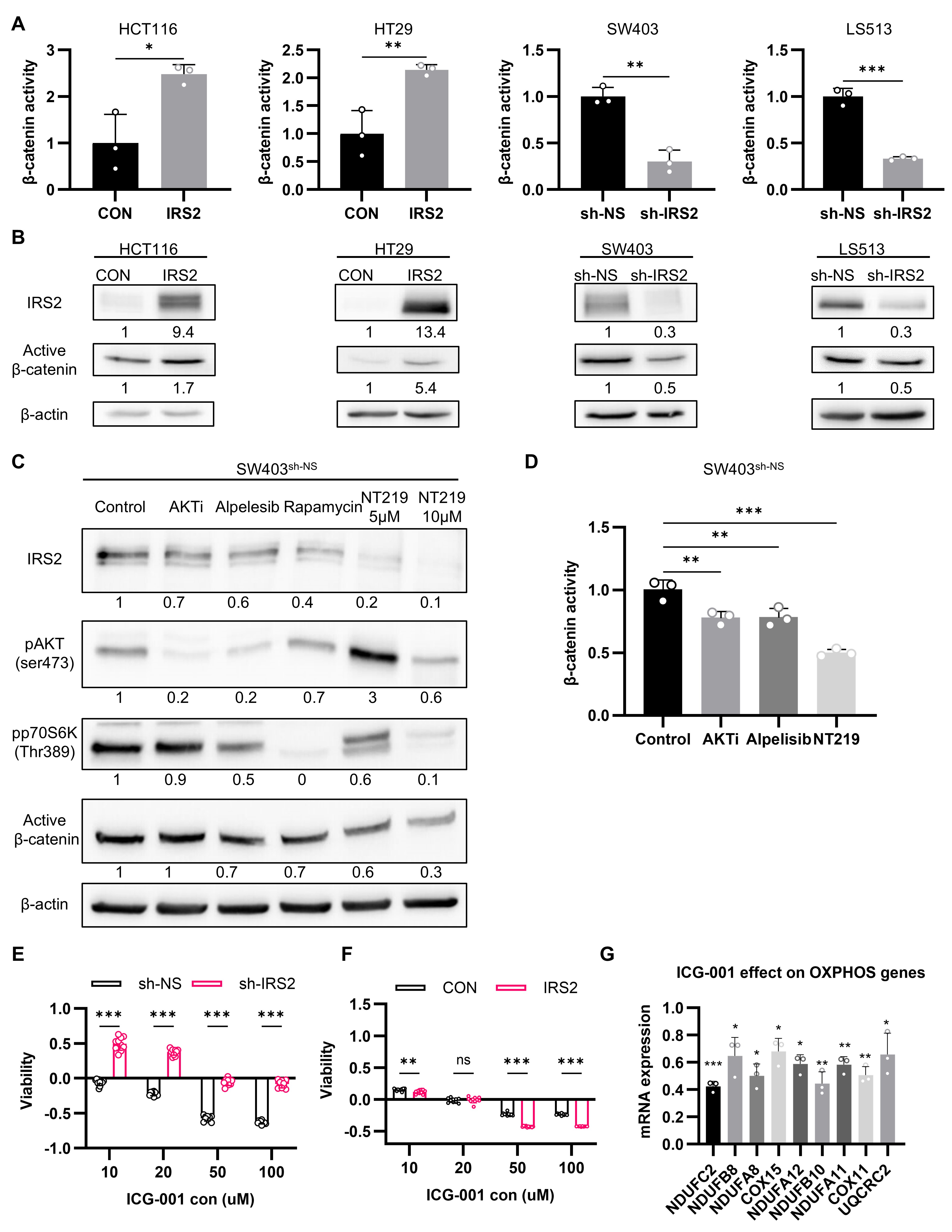

Supplement: noaf028_suppl_Supplementary_Figure_S10 [file noaf028_suppl_supplementary_figure_s10.jpeg]

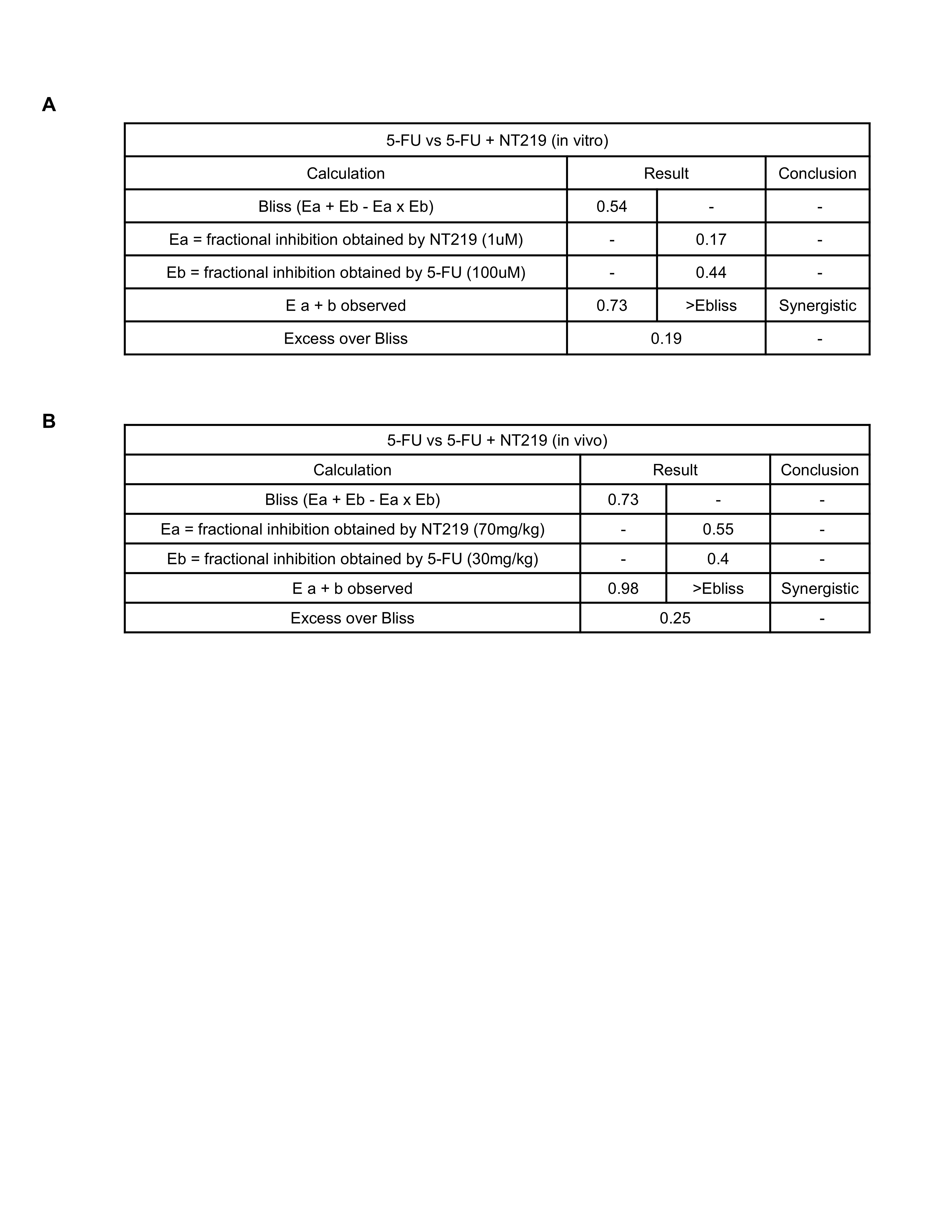

Supplement: noaf028_suppl_Supplementary_Figure_S11 [file noaf028_suppl_supplementary_figure_s11.jpeg]
